# Instillation of a Dry Powder in Nasal Casts: Parameters Influencing the Olfactory Deposition With Uni- and Bi-Directional Devices

**DOI:** 10.3389/fmedt.2022.924501

**Published:** 2022-06-27

**Authors:** Clément Rigaut, Laura Deruyver, Jonathan Goole, Benoît Haut, Pierre Lambert

**Affiliations:** ^1^Transfers, Interfaces and Processes (TIPs), École Polytechnique de Bruxelles, Université Libre de Bruxelles, Brussels, Belgium; ^2^Laboratoire de Pharmacie Galénique et Biopharmacie, Faculté de Pharmacie, Université Libre de Bruxelles, Brussels, Belgium

**Keywords:** nasal cast, nose-to-brain, bidirectional device, nasal spray, powder, artificial mucus

## Abstract

Nose-to-brain delivery is a promising way to reach the central nervous system with therapeutic drugs. However, the location of the olfactory region at the top of the nasal cavity complexifies this route of administration. In this study, we used a 3D-printed replica of a nasal cavity (a so-called “nasal cast”) to reproduce *in vitro* the deposition of a solid powder. We considered two different delivery devices: a unidirectional device generating a classical spray and a bidirectional device that relies on the user expiration. A new artificial mucus also coated the replica. Five parameters were varied to measure their influence on the powder deposition pattern in the olfactory region of the cast: the administration device, the instillation angle and side, the presence of a septum perforation, and the flow rate of possible concomitant inspiration. We found that the unidirectional powder device is more effective in targeting the olfactory zone than the bi-directional device. Also, aiming the spray nozzle directly at the olfactory area is more effective than targeting the center of the nasal valve. Moreover, the choice of the nostril and the presence of a perforation in the septum also significantly influence the olfactory deposition. On the contrary, the inspiratory flow has only a minor effect on the powder outcome. By selecting the more efficient administration device and parameters, 44% of the powder can reach the olfactory region of the nasal cast.

## Introduction

The nasal passage is widely used as a route for drug delivery. The drugs developed for nasal delivery were for a long time only reserved for local treatment (1). But, beyond these local treatments, the nasal route is also a good candidate for systemic delivery. Indeed, this route allows rapid absorption and onset of action that avoids the first-pass metabolism (e.g., gastrointestinal and hepatic pre-systemic metabolism) (2, 3).

Recently, innovative medicines have been developed to use the nose as an entryway to reach the central nervous system by following the path of the olfactory nerve, from the top of the nasal cavity to the brain (1, 4, 5). This route is called “nose-to-brain” (N2B) delivery and aims to deliver the drug to the olfactory region (located at the top of the nasal cavity; Figure 1.3b). Then, the drug diffuses through the olfactory mucosa (Figure 1.4) and subsequently follows the olfactory nerves through the cribriform plate to reach the brain (Figure 1.5). The main advantage of this route is to bypass the blood-brain barrier (BBB), which allows a decrease in the therapeutic dose and thus the side effects as well (6, 7). Moreover, the trigeminal nerve allows reaching the brain without targeting a particular zone of the nasal cavity because this nerve spans over the cavity (8).

**Figure 1:**
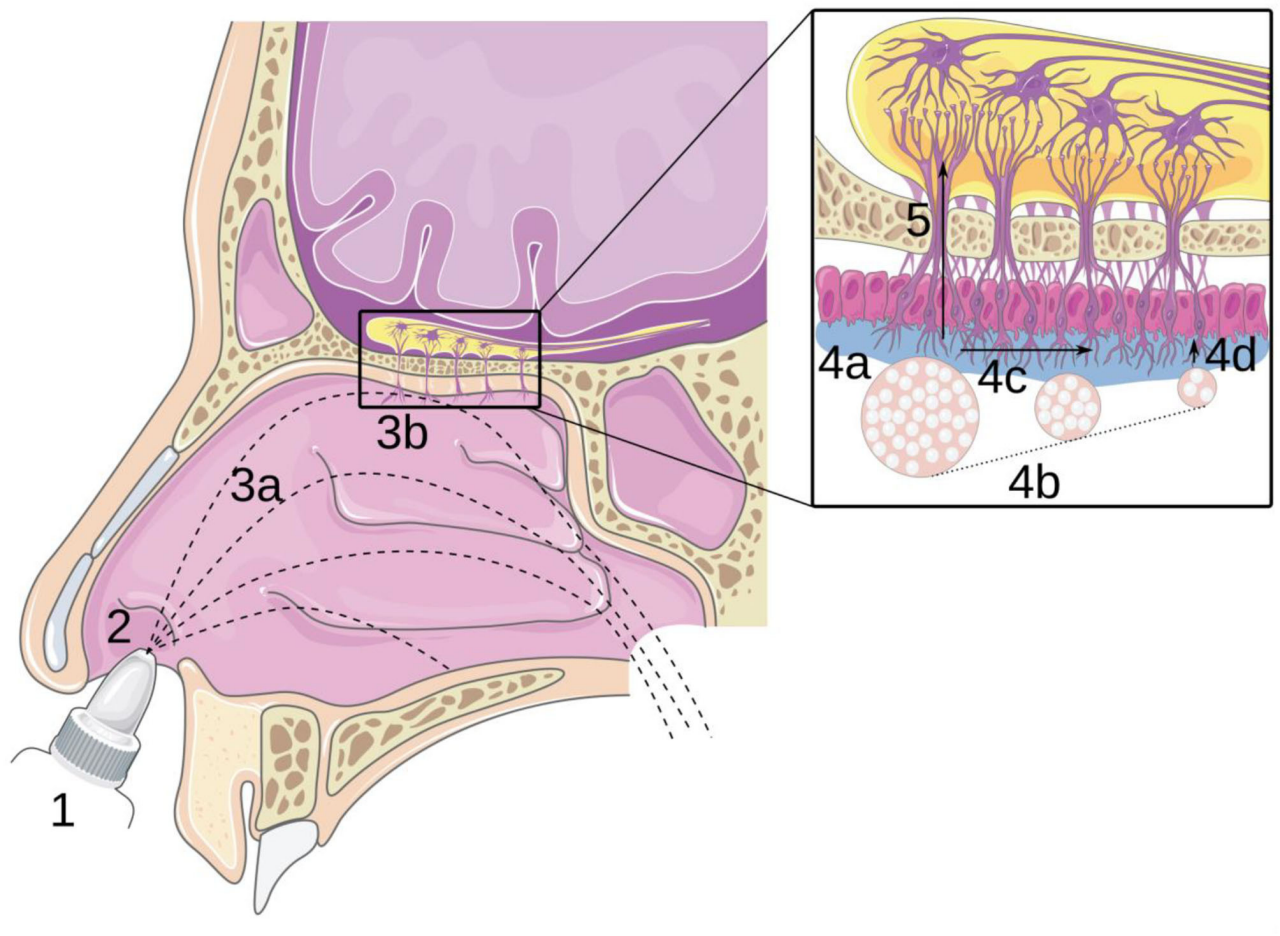
Principle of nose-to-brain delivery: (1) drug formulation; (2) instillation; (3a) transport in the cavity and (3b) impaction of the mucosa; (4) transport in the olfactory mucosa: (4a) adhesion, (4b) dissolution, (4c) mucociliary clearance, and (4d) diffusion; (5) transport through the epithelium and along the olfactory nerve. Reproduced with permission from Ref. (7).

Over the last years, several N2B nasal drugs have already been approved by the FDA, such as Narcan^®^ (naloxone) for opioid addiction (9), Onzetra^®^ Xsail^®^ (sumatriptan) for migraine (10), and Trudhesa™ (dihydroergotamine mesylate) also for migraine (11). The most striking similarity in these products is their rapid onset of action and their formulation, which are all based on a dry powder form. However, the optimization of these treatments is tedious and requires more work to understand the parameters affecting their final efficiency.

Considering the above-mentioned successes of dry powder therapeutics, this work focuses on the deposition of a dry solid model powder (caffeine powder) in the olfactory region of nasal cavities, and the related influencing parameters. We chose to focus on a solid powder because it has been shown that the use of a liquid nasal spray intended for N2B delivery leads to little deposition in the olfactory zone (12–14). For instance, with their Computational Fluid Dynamics (CFD) simulations, Calmet et al. concluded that the olfactory targeting of any liquid nasal spray is insufficient, both with steady inspiration and “sniff” (12). Warnken et al. demonstrated similar results with a maximum of only 2.2% of deposition in the olfactory region of their 3D-printed replica of a nasal cavity (a so-called “nasal cast”) (14). Maaz et al. also reported *in vitro* deposition of about 5% in the olfactory region for liquid sprays. Moreover, their simulations showed that the fraction of the formulation deposited in the olfactory region slightly decreases after the instillation, due to the movement of the liquid after impaction (15). On the other hand, the use of a dry powder seems to be a better choice for N2B delivery. Indeed, Nižić Nodilo et al. studied the deposition of a nasal dry formulation of dexamethasone in a 3D-printed nasal cast. They demonstrated an olfactory deposition efficiency (i.e., a fraction of the instilled powder deposited in the olfactory region) between 5.5 ± 0.9 and 17.0% ± 1.6% (16). Salade et al. also studied the deposition of a liposomal dry formulation in a nasal cast. They demonstrated an olfactory deposition efficiency of 52% (17).

A key factor for olfactory targeting turns out to be particle size. Many studies concluded that the optimal aerodynamic diameter to reach the olfactory region is at most 20 μm to avoid inertial impaction in the nasal vestibule (18–21). On the other hand, this diameter should not fall below 5 μm to prevent the passage of the particles in the lungs (22).

In this work, we focus on the influence of five parameters on the deposition of the model solid powder in nasal cavities: the administration device, the instillation side (left or right nostril), the presence of a septum perforation, the instillation angle and the flow rate of possible concomitant inspiration.

The choice of the administration device is of crucial importance in N2B delivery. The most used devices for nasal drug administration are unidirectional devices generating a classical spray (7, 17). Other administration systems, called bidirectional devices, rely on the expiration of the user: the patient blows into the device to inject the powder into one nostril, causing the soft palate to rise and air to escape through the other nostril (7, 23). The main advantages claimed by these bi-directional devices are a better deposition pattern in the nasal cavity (24, 25) and the avoidance of drug passage in the lower respiratory tract, thanks to the closure of the soft palate (25). Nevertheless, to the best of our knowledge, no study compared these two types of devices for solid powders.

Another important point regarding powder deposition in the olfactory area is the anatomy of the nasal cavity. Indeed, the olfactory area lies at the top of the nasal cavity and the intricate anatomy of the nose restricts the available path to this area (18, 21). Moreover, the anatomy of the nasal cavity can be very different from one person to another, even in healthy patients (14, 26, 27). In addition, some pathologies may change the shape of the nasal cavity and the flow within it [e.g., septal deviation (28–30) or turbinates hypertrophy (29)]. Thus, two important points linked to the anatomy might influence the deposition of a powder in nasal cavities: the insertion side (right or left) and anatomical particularities (hypertrophy or perforation of the nasal septum). In this work, we focus on the influence of an anterior septal perforation on solid powder deposition. Indeed, perforated septums are widespread (1.2% of the worldwide population) and 92% of the perforations are anterior perforations (31). These are known to affect the airflow by increasing the turbulence, creating a recirculation vortex in the perforation, and increasing the velocity immediately downstream (32).

Another parameter that influences the deposition of a solid powder in a nasal cavity is the insertion angle of the device (14, 33–35). Lungare et al. compared the olfactory deposition for different angles with the same insertion depth (5 mm). For one of their formulations, deposition surfaces of 0.91, 0.24, and 0.09 cm^2^ were reported for insertion angles of 60°, 70°, and 80°, respectively (33). Hughes et al. compared the drug deposition in two nasal anatomies with six different angles. The fraction of the instilled powder deposited in the olfactory region varied for each angle condition. It ranged between 0.0 and 0.52% for the first anatomy and between 0.22 and 0.66% for the second anatomy (34).

Finally, the influence of the flow rate of a possible concomitant inspiratory flow on the deposition of a solid powder in the nasal cavity has already been studied (12, 18, 35, 36). Calmet et al. studied the influence of different inspiratory flow conditions on the deposition of various-sized particles. Three inspiratory conditions were studied: sniff condition (A1), steady 20 L/min (A2), and breath-hold (A3; i.e., no inspiration flow rate). For particles with a size of 20 μm, they found an olfactory deposition efficiency of around 0.25, 0.05, and 0% for conditions A1, A2, and A3, respectively (12). Schroeter et al. realized CFD simulations of particle deposition with concomitant inspiration at different flow rates. The fraction of the instilled powder deposited in the olfactory region for particles with a size of 15 μm was 0.0, 4.5, and 2.2% for 7.5, 15, and 30 L/min, respectively (18). These studies highlight the importance of inspiratory flow conditions to the efficiency of the deposition.

To study the influence of these five parameters on the deposition of a solid powder in the nasal cavity, we achieved deposition tests in a 3D-printed nasal cast derived from the CT-Scan. Design-Expert^®^ was used to construct the design of the experiment (DoE) and to reduce the number of tests. For the deposition tests, the use of nasal casts permits realistic and personalized *in vitro* experiments (7, 37). The use of 3D printing allows the creation of a nasal replica for each individual, regardless of their anatomical particularities (37). However, an important difference between a natural nasal cavity and its 3D-printed replicate consists in the adhesion provided by the mucosa. Therefore, the coating of a nasal cast with artificial mucus is needed (38, 39). For instance, Sawant et al. observed an increase in the deposition in their cast from 32 to 38% when artificial mucus was added (39).

## Materials and Methods

### Materials

Caffeine, Kolliphor^®^ P407 (Poloxamer), sodium chloride, potassium chloride, sodium hydrogen phosphate, potassium dihydrogen phosphate, and absolute ethanol (purity ≥ 99%) were purchased from Sigma-Aldrich (St Louis, MO, USA). Milli-Q (ELGA) water (>18.2 MΩ cm, 21°C) was used as a solvent.

### Model Powder

Caffeine powder was used to perform the deposition tests. Caffeine is readily soluble in water (21.6 mg/ml) (40) and ethanol (15.15 mg/ml) (41) and it is easily quantifiable by spectrophotometry-UV (42, 43).

#### Quantitative Caffeine Determination

The quantitative determination of the caffeine concentration in a solution of ethanol by spectrophotometry-UV was adapted from Ahmad Bhawani et al. (44). Briefly, a standard stock solution (0.1 mg/ml) was prepared by dissolving 10.0 mg of caffeine in 100.0 ml of ethanol absolute. In addition, the stock solution was diluted with absolute ethanol to give five different concentrations of caffeine (50.0, 20.0, 8.0, 4.0, and 2.0 μg/ml), which were used in the linearity study. Each standard stock solution was analyzed at 274 nm using UV/Vis spectrophotometry (Implen NanoPhotometer^®^).

#### Particle Size Characterization

The caffeine was sifted through a 0.123 mm sieve to deagglomerate and have a particle diameter closer to the ideal size for N2B delivery (median diameter smaller than 20 μm). The sieve of 0.123 mm was used because it was found to give the best results in terms of particle diameter.

The aerodynamic particle size distribution at the exit of the devices used in this work was determined by laser diffraction using a Spraytec™ apparatus in an open bench system with a 100 mm lens (Malvern Panalytical, Malvern, UK). The analysis parameters were set as follows: test duration of 300 ms, actuation distance of 7 cm, and data acquisition rate of 2,500 Hz. Data are expressed as Dv10, Dv50, and Dv90 and span values. The particle size distribution at the exit of the devices was measured in triplicate, for both the unidirectional and bidirectional devices.

### Uni- and Bidirectional Devices

#### Device Characterization

**Figure 2:**
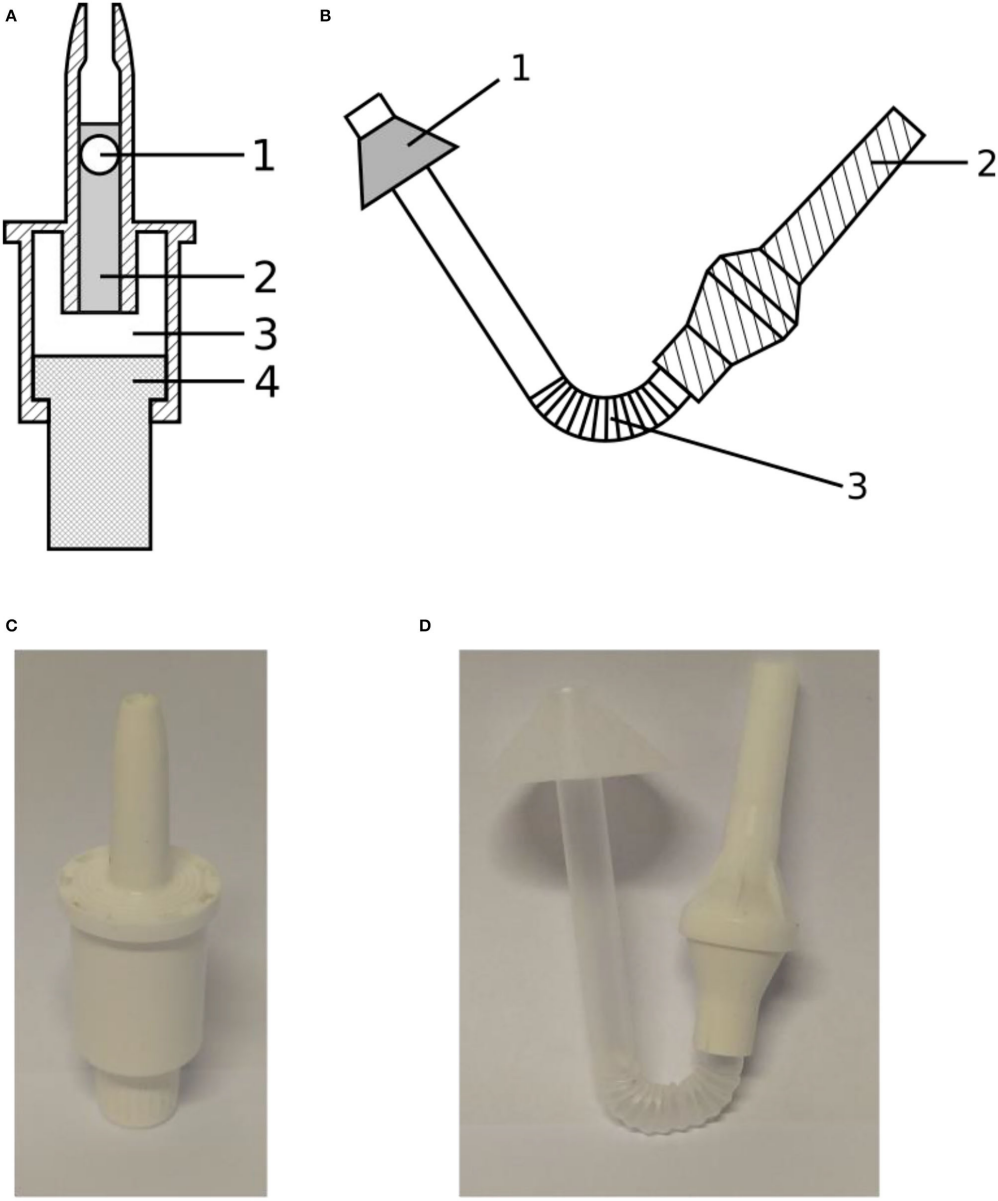
**(A)** Schematic drawing of the Aptar UDS unidirectional device showing (1) the sealing bead, (2) the cylinder containing the powdered formulation, (3) the reservoir containing propulsion air, and (4) the piston compressing the air; **(B)** Schematic drawing of the IP Med TriVair bidirectional device showing (1) the nosepiece, (2) the mouthpiece, and (3) the section containing the powdered formulation; **(C)** Picture of the Aptar device; **(D)** Picture of the IP Med device.

In this study, two types of nasal delivery devices were used: a unidirectional device producing a solid cone (UDS, Aptar, Le Vaudreuil, France), which was designed specifically to maximize the amount of drug reaching the olfactory zone, and an innovative bidirectional device (TriVair™, IP Med Inc., Oceanside, NY, USA) to compare their effectiveness, measured as their ability to deliver a dry powder to the olfactory region. These two devices are illustrated in Figure 2.

The unidirectional powder spray is a ready-to-use single-use device. A cylinder (Figure 2A.2), closed by a bead (Figure 2A.1), contains the powder. A piston (Figure 2A.4) compresses the air of an internal chamber (Figure 2A.3) to inject the powder through the nozzle. The maximum filling volume is 140 mm^3^. Its advantages are its high ejection velocity and patient-independent performance. The bidirectional device is made of a pipe connecting the mouth and the nose to inject the powder into the nasal cavity. The powder, laying in the curved section of the device (Figure 2B.3) is ejected when the patient blows through the mouthpiece (Figure 2B.2). A cone, on the nasal part of the pipe, is meant to provide an airtight seal between the device and the nostril. The main advantage described for this bidirectional device is that the soft palate raises during a forced expiration by the mouth, preventing any powder deposition in the lower respiratory tract.

#### Plume Angle and Ejection Velocity

The plume angle and the ejection velocity of the administration devices were determined by high-speed imaging. The camera is an IDT motion pro Y3 (Integrated Design Tools, Pasadena, CA, USA) with a Nikon AF micro-Nikkor 60 mm f/2.8 D lens (Nikon, Tokyo, Japan). The imaging parameters were an aperture of 16, an acquisition time of 100 μs, and an acquisition frequency of 3,000 Hz. The tests were done in triplicate for each device.

Then, an original Python script using the scikit-image library v. 0.19.1 processed the images to obtain the plume angle and the ejection velocity. For the speed, the front of the plume at the beginning of the actuation was tracked across multiple images. By dividing the advance of the plume front between two frames by the time elapsed, we obtained the ejection velocity. For the plume angle, the background was first extracted, and the images were transformed into black-and-white pictures. A Sobel algorithm permitted plume detection followed by a Hough filter to detect the edges corresponding to the plume and to measure the angle.

### Nasal Cast

#### Choice of the Patient

**Figure 3:**
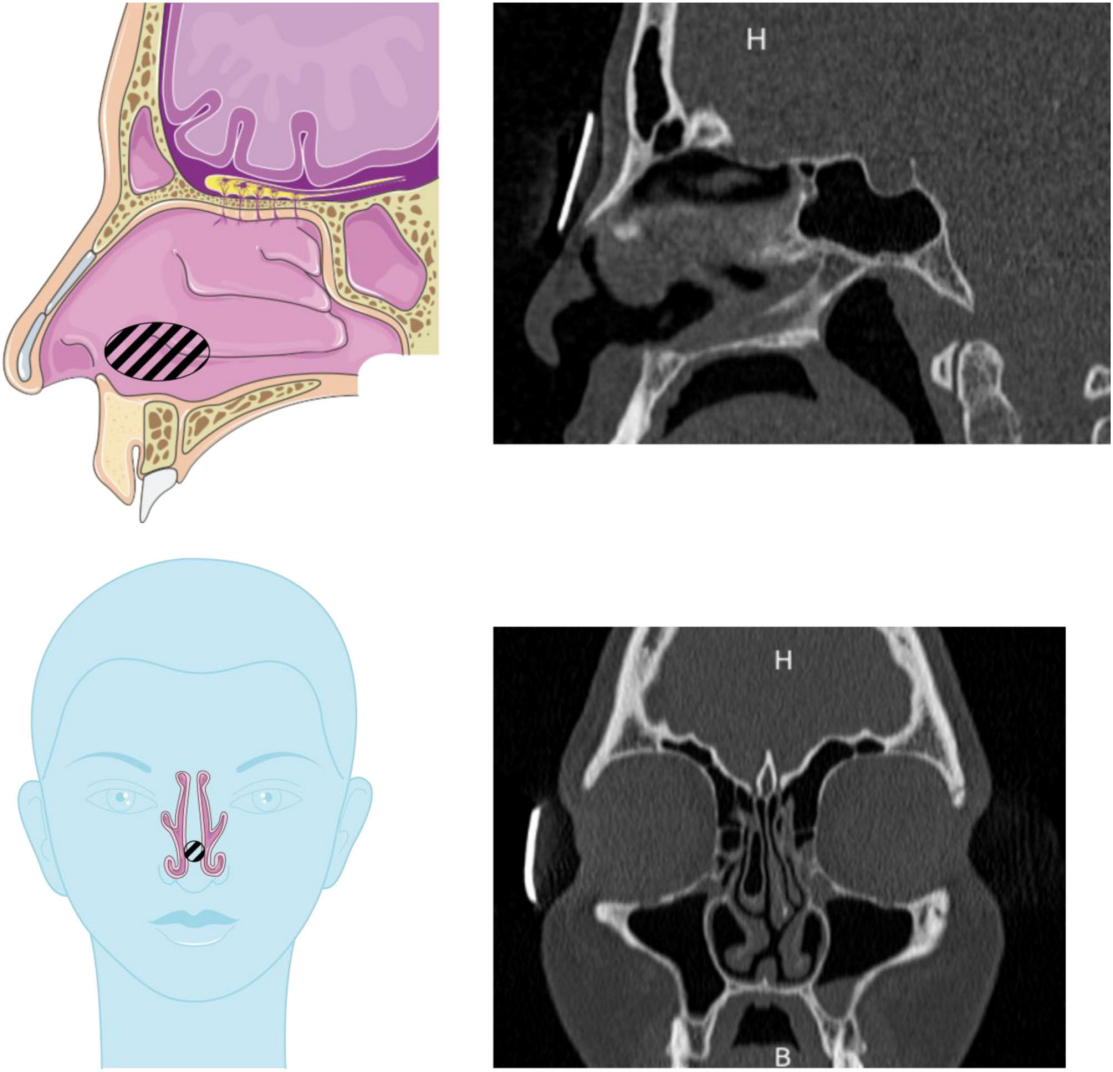
Drawings and scans showing the septum perforation in the nasal cavity used in this study. The septal perforation is hatched on the drawings. Drawings adapted from the Servier Medical Art.

The patient selected for this study is an adult with a septum perforation. However, the rest of the anatomy was normal: no other anatomical disorder such as major septum deviation, nasal polyps, or hypertrophied turbinates. Therefore, the results obtained with the nasal cast derived from this patient are expected to be representative of “normal” anatomy (except for the septum perforation). Consequently, two different geometries were used in this study: the original one with the septum perforation and a “healthy” geometry, constructed by artificially filling the hole in the septum. Figure 3 shows the location of the septum perforation (hatched area) in the nasal cavity.

#### Creation of the 3D Model From a CT-Scan

For obtaining a nasal cast with high resolution, it has been derived from the CT scan of the nasal cavity of the above-mentioned patient. First, each grayscale image composing the CT scan was binarized, *via* the choice of a threshold (45), to separate the airways (in white) from the body (in black). However, to obtain printable parts, a post-processing step was needed to avoid the presence of white pixels in the body or black pixels in the airways. This treatment was done using two morphological operations: a closing with a square 3 × 3 structuring element, followed by an opening, with the same structuring element. The final step in the image treatment was to remove the sinuses from the geometry. Indeed, N2B delivery is studied in this work and the sinuses were shown to have no impact on the drug deposition when such a route of administration is targeted (26). The images were also cropped to keep only the relevant part to reconstruct the nasal passages.

Once the original CT scan was transformed into black-and-white images, the 3D geometry of the nose was reconstructed using InVesalius software v. 3.1.1 (Centro de Tecnologia da Informação Renato Archer, Campinas, Brazil). However, this model was not representative of the biological cavities yet. Indeed, the resolution of the scans being 0.3 mm in the horizontal direction and 0.7 mm in the vertical direction, the 3D reconstruction led to stair-like surfaces. Therefore, the final step before obtaining a usable geometry was to smooth the surface *via* a recursive Lagrangian smoothing in Meshlab software v 2021.05 (Istituto di Scienza e Tecnologie dell Informazione, Pisa, Italy). This smoothing was stopped when the ratio surface/volume between two smoothing iterations did not evolve more than 1%.

#### Nasal Cast Design

**Figure 4:**
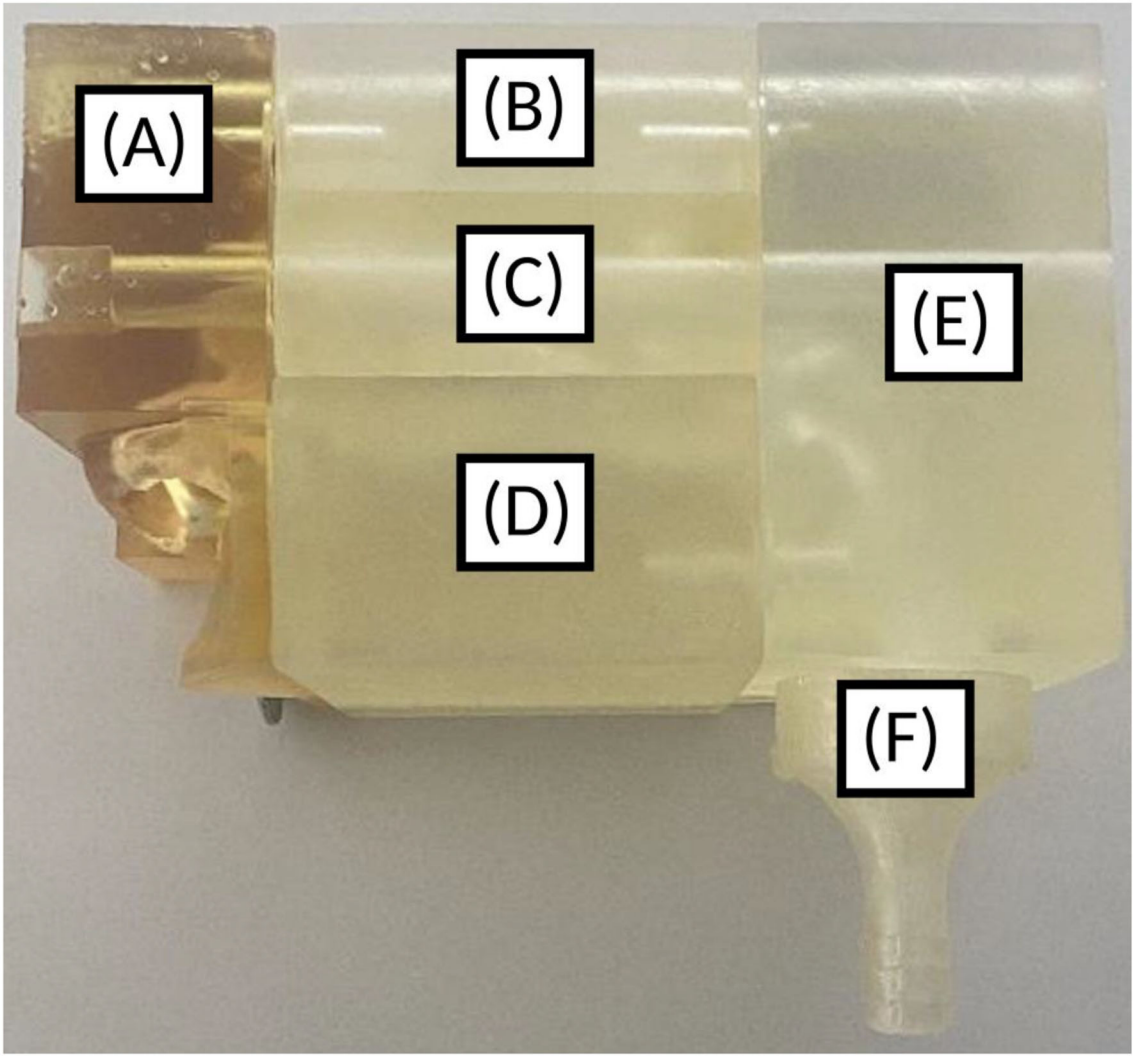
3D-printed nasal cast. **(A)** Nostril, **(B)** Olfactory region, **(C)** Middle turbinates region, **(D)** Lower turbinates region, **(E)** Nasopharynx, **(F)** Post-nasal fraction.

Once the 3D geometry of the nasal cavity was obtained, the nasal cast itself was designed using FreeCAD v. 0.19.1. The positive of the nose was cut into five pieces. These pieces correspond to the nostrils, lower turbinates, middle turbinates, olfactory region, and nasopharynx (Figure 4). The olfactory region was defined as the walls enclosing the superior turbinate and meatus and the corresponding segment of the nasal septum.

An adaptor for a pump was also added at the end of the nasopharynx to possibly simulate an inspiration concomitant to the instillation. A filter was placed between the cast and the pump tubing to measure the post-nasal fraction (i.e., the fraction of the instilled powder that does not deposit in the nasal cavity). There was also a possibility to add a cap to simulate the closing of the soft palate. In addition, the cast integrated spaces for joints to provide an airtight seal between the pieces, especially when using aspiration. Two casts were printed: one with the septum perforation and another one with this perforation previously filled on the 3D geometry.

### Mucus Coating

#### Artificial Mucus Fabrication

A thermosensitive gel was developed to have a thin adherent layer of mucus on the nasal cast. Briefly, 25% w/w of Poloxamer^®^ 407 (46) was added to a simulated nasal electrolyte solution (SNES) (47). This gel is liquid under 10°C and swells around 18°C. Thus, the artificial mucus was applied cold in the nasal cast with a micropipette.

#### Adhesive Strength of the Artificial Mucus

The adhesive strength of the thermogel was evaluated by comparing the amount of powder sticking to the mucus under a moderate airflow (15 L/min) and the amount of powder sticking under a strong airflow (100 L/min) (48). A sample of mucus with the powder was placed on the top of a curved tube having the same hydraulic diameter as the nasal cavity in the middle of the turbinates. Two valves allowed us to change the direction of the flow, simulating a respiration cycle. The duration of each phase of the cycle (inspiration or expiration) has been set to 5 s.

#### Uniformity of Mucus Coating

The incorporation of caffeine in the thermosensitive gel allowed analyzing the uniformity of the mucus deposition. A thermosensitive gel with 1.0 mg/mL of caffeine was prepared as previously described. Then, each part of the nasal cast was cooled to −8°C before coating it with mucus using a micropipette.

After gelation, the cast was disassembled, and each of its five parts was rinsed separately with ethanol absolute. The amount of caffeine in each of the six solutions was evaluated by UV spectrophotometry. Such data provided the volume of mucus coated in each part of the cast. By dividing this volume by the surface of the piece, an estimate of the mucus thickness can be obtained to assess the coating uniformity. Each test was done in triplicate and analyzed with standard deviation and variation (mean/SD).

### Study of the Powder Deposition

#### Deposition Tests

The nasal cast was cooled to −8°C for 10 min before coating with the artificial mucus. After coating, the nasal cast was left at ambient temperature for 10 min for the thermosensitive gel to jellify. The different parts of the nasal cast were assembled before the powder instillation. The inspiratory flow rate (L/min) was fixed by using a DFM3 flow meter (Copley Scientific, Nottingham, UK) and was obtained with two HCP5 air pumps (Copley Scientific) connected in series to a TPK critical flow controller (Copley Scientific). The flow used in the experiments is a steady flow. The pumps were turned on at least 5 s before the instillation to ensure that the steady-state regime was reached and turned off at least 10 s after the instillation to ensure that all the powder was deposited before stopping the flow.

The mass of the powder introduced in the device ranged between 20.2 and 23.5 mg. We fixed this dose because 50 mg is considered the maximum dose for nasal delivery (15, 49) (25 mg per nostril and per shot). The devices were weighed before and after the instillation in the nasal cast to know the exact injected dose. We calculated the percentage of powder recovery with the following equation:


(1) $$\begin{array}{r} \%recovery=\frac{\left(mass\;of\;the\;device\;before-mass\;of\;the\;device\;after\right)}{total\;mass\;of\;powder\;recovered\;in\;the\;nasal\;cast}\% \end{array}$$

Each run with a percentage of recovery lower than 70% was rejected and repeated. The position of the device was controlled by 3D-printed supports ensuring to have the same spray location and angle for each repetition of a given test.

The actuation of the unidirectional device was realized by hand (because of its patient-independent performance) and the actuation of the bidirectional device was realized with a blowing bulb to mimic an expiration (140 ± 23 L/min) (50).

Then, the nasal replica was disassembled, and each of its five parts and the filter was rinsed with ethanol absolute. Finally, the caffeine concentration in each solution thus obtained was measured at 274 nm using UV spectrophotometry, as previously described, and was converted into the fraction of the instilled powder deposited in the corresponding part of the cast.

#### Design of Experiment (DoE)

Several factors that influence the deposition in nasal casts have already been reported in the literature: the emitted volume, the plume geometry, the particle or droplet size, the head orientation, the administration angle, the nozzle insertion depth, the breathing profile, and the anatomy (51). In this work, five factors were selected, each one having two or three design points. The first is the insertion angle of the device. Two different angles were fixed: one targeting the center of the nasal valve (referred to as “center”) and the other one corresponding to a direct sighting of the olfactory region (referred to as “direct aim”). These two angles were selected because they can be directly measured on the patient's anatomy and not on an external axis and because both were reported to increase the deposition beyond the nasal valve (52). As mentioned in the introduction, the second factor is the presence or absence of a perforation of the septum. Thus, the two design points are this slight anatomical variation: with and without septum perforation. The third factor is the side of instillation: the left or right nostril. The nostril in which the instillation is done can influence the deposition pattern: Frank et al. (29) observed that 18% of a spray of 20 μm droplets passed the nasal valve on one side of the studied nasal cavity but only 3% on the other side. The fourth factor is the delivery device itself, and the fifth factor is the flow rate of a concomitant inspiration. For that, three levels were fixed: without inspiration (0 L/min), breathing at rest (15 L/min), and high inspiratory flow (60 L/min), to simulate the sniff reflex that the patient may have when receiving a spray (12). This last factor is only used with the unidirectional device since the bidirectional device prevents any inspiration.

In this work, three different designs of experiments (DoE) were performed with the Design-Expert^®^ software (Version 13, Stat-Ease Inc., MN, USA). The selected design was the factorial randomized optimal one. The first DoE is called the “full DoE.” It includes all factors, except the inspiratory flow. The second DoE allows evaluating the influence of all factors (angle, inspiratory flow, perforation, and side) with the unidirectional device. Please also note that the instructions of use by Aptar recommended avoiding concomitant inspiration. Finally, the third DoE studies the influence of different factors (the angle, the perforation, and the side of instillation) with the bidirectional device.

Six different responses for each DoE were selected. These are the fractions of the instilled powder deposited in the five parts of the nasal cast, plus the post-nasal fraction (14, 53). The results of each DoE were analyzed using analysis of variance (ANOVA). A *p*-value of <0.05 was considered statistically significant.

## Results and Discussion

### Powder Characterization

As previously described, the optimum particle size for the olfactory deposition is lower than 20 μm (21). Table 1 shows that the volume median diameter (Dv50) is smaller for the unidirectional device than for the bidirectional one (25.21 ± 1.45 μm and 69.79 ± 12.2 μm for the unidirectional and the bidirectional device, respectively). It seems to indicate that the unidirectional device can deagglomerate the powder more efficiently than the bidirectional device. Consequently, better deposition is expected for the unidirectional device due to particle size distribution that is more adapted for N2B delivery.

**Table 1:** Powder characteristics.

	**Dv10**	**Dv50**	**Dv90 (μm)**	**Span**
	**(μm)**	**(μm)**		
Unidirectional device	10.38 ± 0.53	25.21 ± 1.45	54.46 ± 3.05	1.76 ± 0.03
Bidirectional device	24.25 ± 5.07	69.79 ± 12.20	159.84 ± 44.97	1.89 ± 0.27

*Each result is expressed as mean ± SD*.

### Plume Angle and Ejection Velocity

The plume angle is bigger from the unidirectional device than that from the bidirectional device. It reaches 16.8° ± 2.4° and 3.9° ± 0.3° for the unidirectional device and the bidirectional device, respectively. As already described in the literature, a device that produces a smaller plume angle increases the deposition beyond the nasal valve due to the reduced impaction (35, 54, 55). Nevertheless, both devices are characterized by a narrow plume angle, which should allow targeting of the post-nasal-valve region. Indeed, Foo et al. described an efficient deposition (about 90%) in the post-nasal-valve region when the plume angle was lower than 30° (35). Note that, contrary to liquid devices, where the plume angle can be controlled by the viscosity of the formulation (35, 54), the plume angle of a powder device is almost exclusively controlled by the device itself.

The ejection velocity from the unidirectional device is almost 10 times higher than the ejection velocity from the bidirectional device. It reaches 49.2 ± 6.0 m/s and 5.9 ± 0.1 m/s for the unidirectional and the bidirectional devices, respectively. Using these values and if we assume spherical particles, we can calculate the drag coefficients *c*_*d*_ of the solid particles using the following equation (56):


(2) $$\begin{array}{r} c_{d}=\frac{24}{Re}\left(1+0.1935Re^{0.6305}\;\right) \end{array}$$

where *Re* is the Reynolds number of the particle at the exit of the administration device. This equation is valid for Reynolds numbers between 20 and 260, which is the case in our study. Indeed, it can be computed with the following equation:


(3) $$\begin{array}{r} Re=\frac{uL}{\nu } \end{array}$$

where *u* is the relative speed of the particle in the air, *L* is the diameter of the particle, and ν is the kinematic viscosity of the air (taken equal to 12.24 10^−6^ m^2^/s). It gives a Reynolds number of 27 for particles exiting in the bidirectional device and 82 for particles exiting in the unidirectional one. Thus, drag coefficients equal to 1.10 and 2.02 are calculated for the unidirectional and the bidirectional devices, respectively. Therefore, the particle trajectories are less influenced by air friction with the unidirectional device than with the bidirectional one since their drag coefficient is lower. A better deposition in the olfactory region may thus be expected using the unidirectional device. Moreover, the airflow used to eject the powder from the bidirectional device is 140 ± 23 L/min, which is similar to the normal expiratory flow rate (50). In addition, the duration of the airflow meant to empty the device is also sufficient to eject all the powder.

### Mucus Coating

#### Uniformity of the Mucus Coating

Before the deposition experiments, the uniformity and the thickness of the mucus coating were checked. The low SD and percentage of the variation of each region of the nasal cast indicate that the mucus deposition can be repeated (Table 2). The approximate thickness of the mucus showed that the mucus layer was uniform across all the parts.

**Table 2:** Results of the experiment of the mucus coating uniformity (*n* = 3).

**Region**	**Mean of caffeine**	**SD**	**Variation**	Thickness^*^
	**deposition (μg)**	**(μg)**	**(%)**	**(μm)**
Nasopharynx	194.29	2.70	1.39	57.11 ± 0.79
Olfactory region	146.04	2.08	1.42	80.16 ± 1.14
Lower turbinates	562.99	5.97	1.06	57.25 ± 0.61
Middle turbinate	343.86	2.05	0.60	57.65 ± 0.34
Nostrils	121.99	0.46	0.38	35.86 ± 0.14

* *Thickness is expressed as mean ± SD*.

#### Adhesive Strength of the Mucus

**Figure 5:**
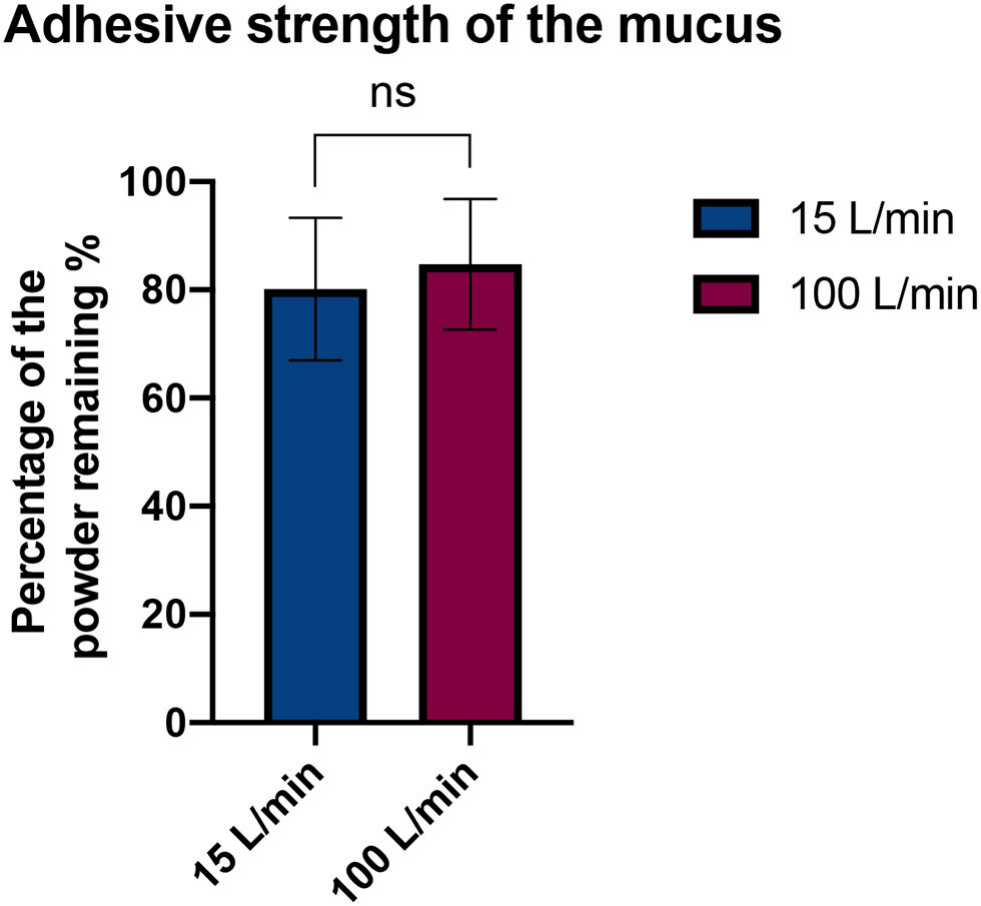
Comparison of the adhesive strength of the mucus with two flow rates. Results are expressed as mean ± SD. Student's *t*-test does not reveal a significant difference (*p*-value = 0.4353).

No significant difference was observed between the two airflows (15 and 100 L/min) as the percentage of powder stuck on the mucus remained constant after 10 respiratory cycles (Figure 5). Therefore, the artificial mucus was considered adherent enough for our tests.

### Design of Experiments

For each DoE, a linear model with interactions was fitted. In all cases, all the factors were included along with their significant interactions. A parameter is considered significant if its *p*-value is lower than 0.05. The non-significant interactions are not included if they are not mandatory to get a hierarchical model. The three ANOVA tables (Supplementary Tables 4–6) corresponding to the three DoE are presented. The detailed effects of each parameter are described in the rest of this section. The mean percentage of the powder recovery in our tests was 86.0% ± 7.2% (computed with the formula Eq. 1).

#### Device

**Figure 6:**
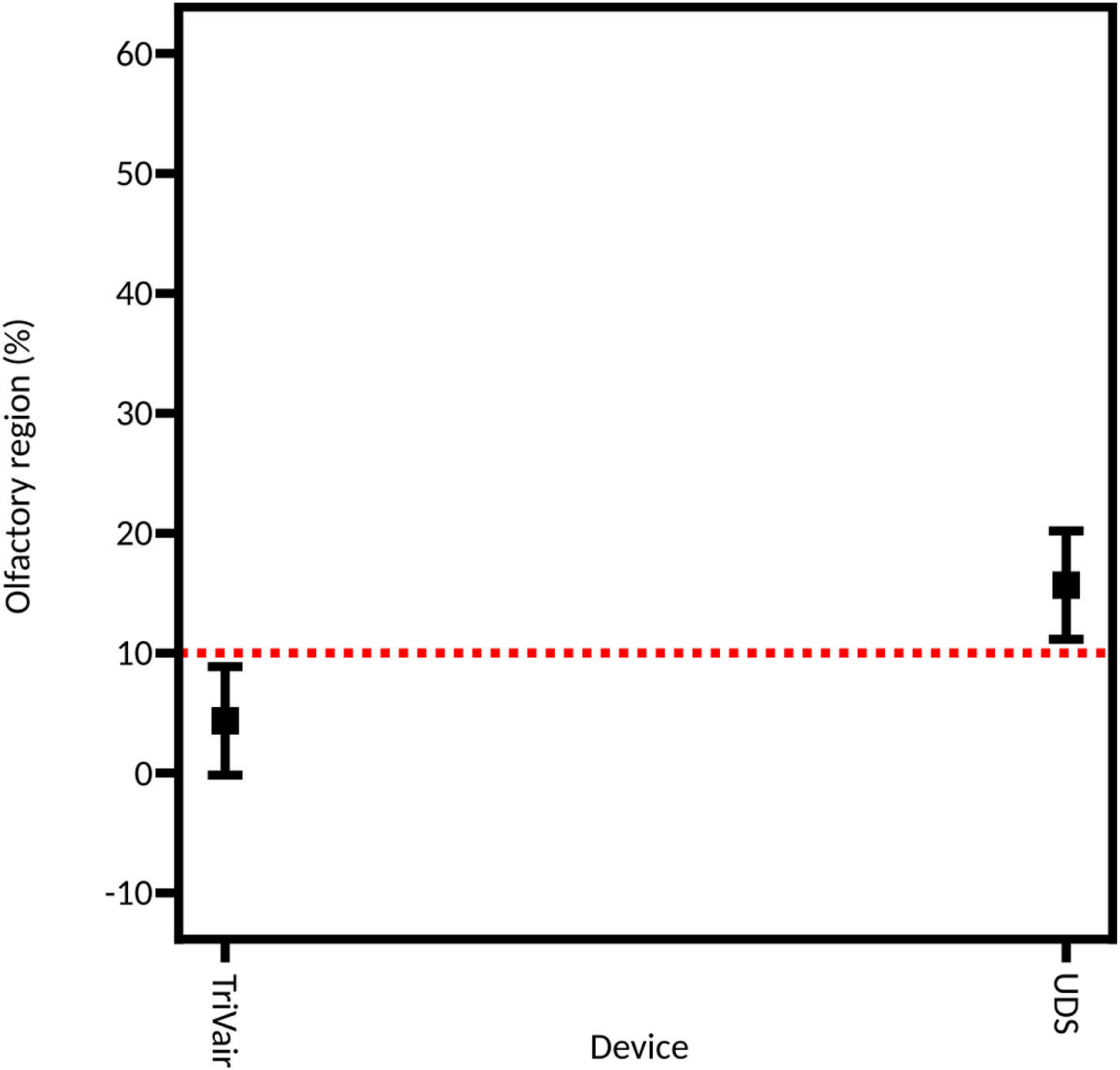
Model graph showing the influence of the device on the powder deposition in the olfactory region. This model is extracted from the full DoE. Results are expressed as mean ± RMSE.

As described above, two different technologies were compared: unidirectional and bidirectional devices. Figure 6 shows that the unidirectional device is more efficient for depositing powder in the olfactory region (22.33% ± 4.86% of the powder deposited in the olfactory region on average) than the bidirectional device (7.11% ± 1.52% of powder deposition on average). A previous study compared the difference in nasal deposition between a liquid nasal spray and a bidirectional powder device. Djupesland et al. demonstrated a higher deposition in the upper region of the nasal cavity with the bidirectional device compared to the liquid nasal spray (18.3% vs. 2.4% for the bidirectional device and the liquid nasal spray, respectively) (57). In our study, olfactory deposition is higher with the unidirectional device than with the bidirectional device. The difference lies probably in the use of a solid powder instead of a liquid and, in particular, in the size of the particles. Indeed, previous studies used the same particle size for both types of devices (58) while, in our study, the unidirectional device was more efficient to deagglomerate the powder, resulting in a more suitable particle size distribution.

In a recent study, Lapidot et al. studied the deposition of a powder of Naloxone in a nasal cast. They also used the UDS Aptar device (unidirectional device). They showed an olfactory deposition of 51% despite a volume median of particles of 77.7 μm (59). Thus, we can expect another influencing parameter than the particle size distribution to explain our better deposition with the unidirectional device.

#### Insertion Angle

**Figure 7:**
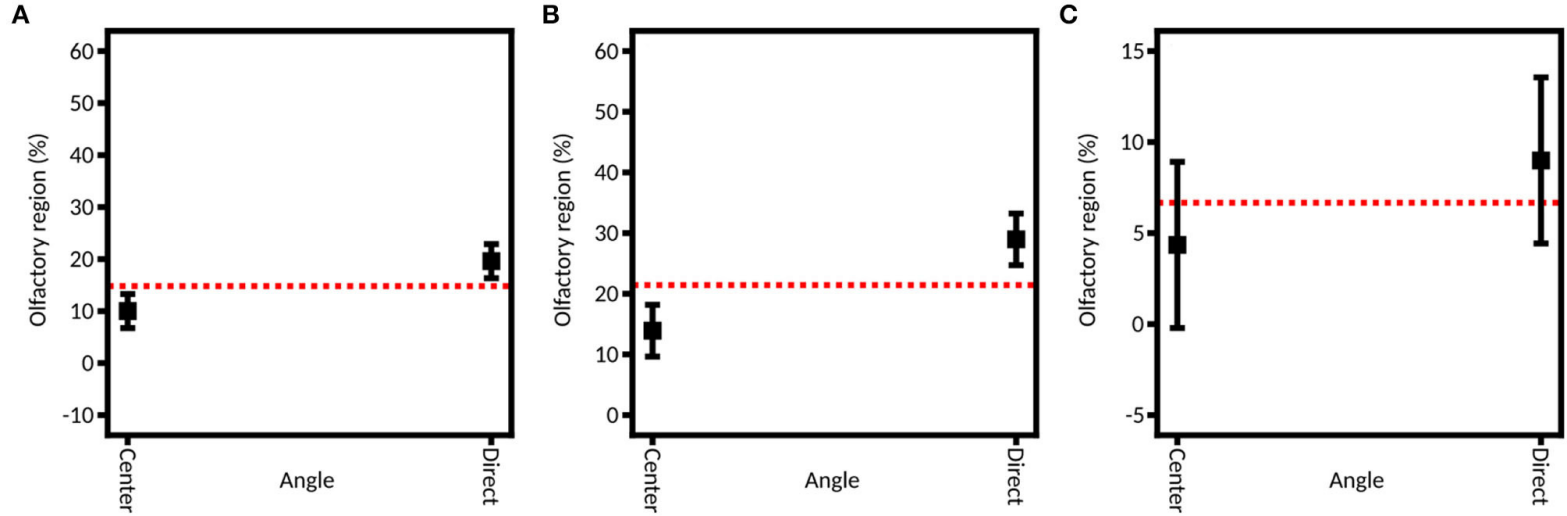
Model graph of the angle influence in olfactory deposition from **(A)** full DoE, **(B)** DoE focused on the unidirectional device, and **(C)** DoE focused on the bidirectional device. Results are expressed as mean ± RMSE.

Figure 7 shows the linear models fitted on each of the three DoE. It appears that the direct aim of the olfactory region leads to a better deposition in the olfactory zone (19.78% ± 5.64%) than the aim at the center of the nasal valve (9.67% ± 3.12%). If the two devices are tested separately, we can see that this influence is only significant with the unidirectional device (28.79% ± 3.65% for direct aim vs. 12.47% ± 3.81% for the aim at the center of the valve) and not the bidirectional one (8.88% ± 4.35% vs. 4.35% ± 1.86%).

As previously described, our study is based on the work of Tong et al. (52) and Basu et al. (60) to select these angles. Each team respectively recommended aiming at the center of the nasal valve or directly at the olfactory zone. However, they used liquid nasal sprays. Tong et al. demonstrated that to avoid the nasal valve barrier, a solution is to aim to the center of the valve to get a spray nozzle – nasal valve alignment and to improve nasal deposition in the depth of the nasal cavity (52). Basu et al. (60) demonstrated that to reach a selected zone, the best practice is to follow the direct line of sight. Our results show that this second option increases by about 10% the olfactory deposition. This could be explained by the fact that the powder devices produce narrower plumes than the liquid sprays used by Tong et al. (50° in their case and 4° to 17° in our case). Therefore, there was less impaction on the nasal valve, so this anatomic part obstructed less of the access to the posterior region of the cavity than the larger spray plumes. Those results are in line with a recent review by Maaz et al. (15), which shows that all studies agree on the fact that wide plumes (larger than 30°) are unable to reach efficiently the turbinates.

#### Instillation Side

**Figure 8:**
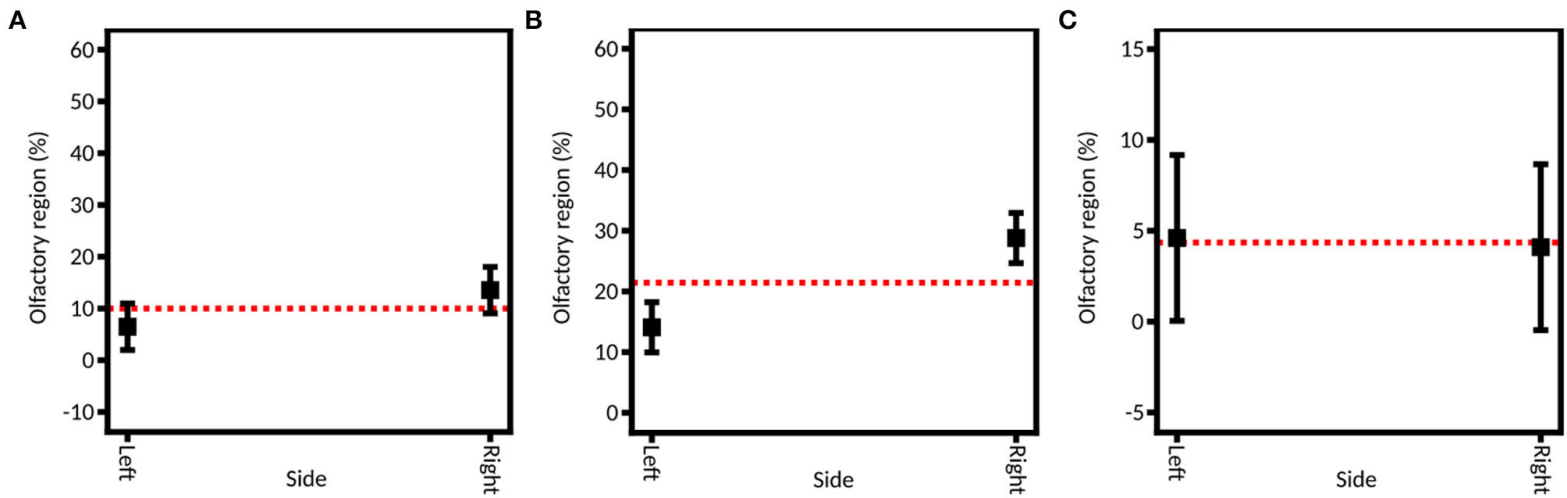
Model graph of the instillation side influence in olfactory deposition from **(A)** full DoE, **(B)** DoE focused on unidirectional device, and **(C)** DoE focused on bidirectional device. Results are expressed as mean ± RMSE.

To assess the role of the injection nostril, caffeine was instilled in both nostrils, using both devices. A significant influence of the injection nostril is found from the full DoE (average 20.33% ± 5.46% on the right side and average 9.12% ± 1.68% on the left side) and the DoE focused on the unidirectional device (27.70% ± 3.94% on the right and 13.56% ± 3.01% on the left; Figure 8). That could be explained by the fact that the right olfactory region is larger than the left one: the left side and the right side were characterized by a surface of 10.5 and 11.4 cm^2^, respectively. Thus, more powder can deposit on the right side. Consequently, this element should be a decision guide when choosing to inject a drug into one nostril or the other if personalized medicine is sought. However, for the bidirectional device, no significant difference is observed between the two sides (6.82% ± 2.41% on the right and 6.93% ± 2.38% on the left). It may be explained by the higher particle velocity (49.2 ± 6.0 m/s) that is observed with the unidirectional device, leading to deeper deposition in the olfactory area. On the other hand, the deposition with the bidirectional apparatus is shallower (velocity of 5.9 ± 0.1 m/s) and thus not affected by the administration nostril.

Let us also mention that the two nasal valves' cross-section areas differ strongly: the left one is 113 mm^2^ and the right one is only 75 mm^2^. However, as mentioned previously, the spray plumes used are narrow. So, in our case, the nasal valve is not an obstacle to effective powder deposition in the turbinates.

#### Perforation

**Figure 9:**
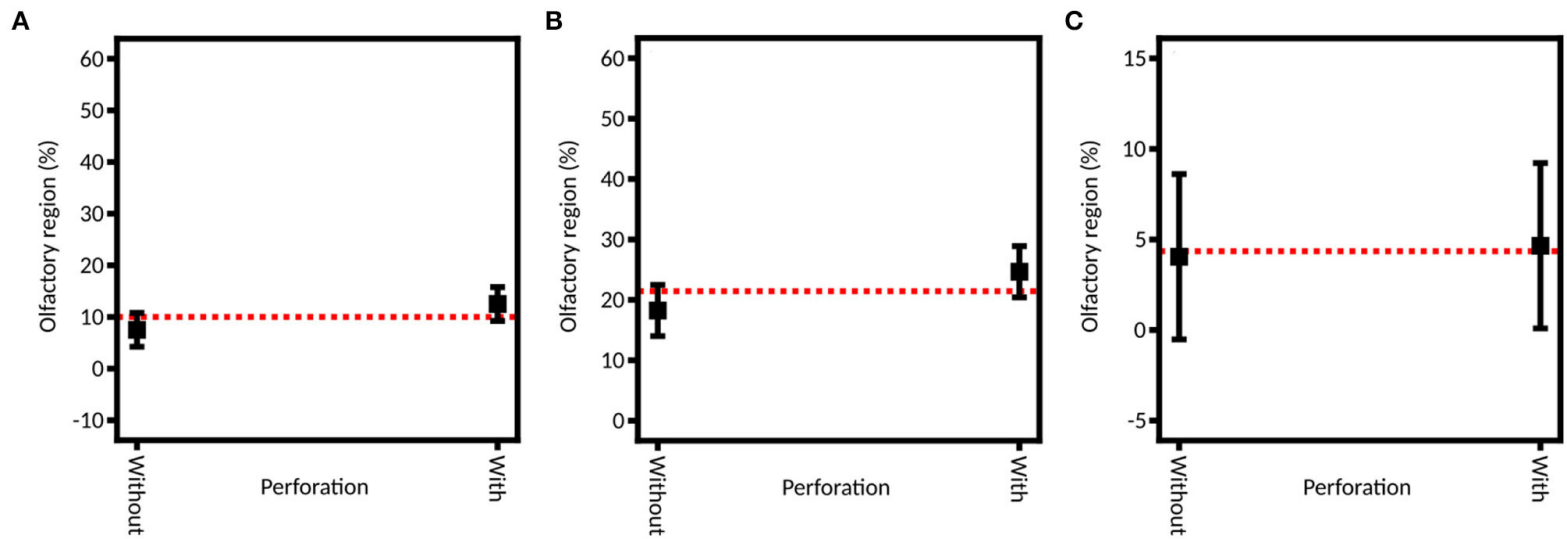
Model graph of the influence of the septum perforation in olfactory deposition from **(A)** full DoE, **(B)** DoE focused on unidirectional device and **(C)** DoE focused on bidirectional device. Results are expressed as mean ± RMSE.

By selecting an example of perforation, the influence of a healthy (no septum perforation) and a pathologic (with septum perforation) state on the spray distribution was studied (Figure 9). No significant difference is observed between these two states for the olfactory region deposition, neither for the full DoE nor for the two subsequent ones (*p*-values of 0.1626 when using indifferently both devices, 0.3010 and 0.9345 when using the unidirectional and bidirectional devices, respectively). This result can seem surprising given the large impact that septum perforation has on nasal airflow (32, 61). However, in the case of a lower-anterior perforation (like in this study), the change in the flow does not propagate above the lower turbinate (61). Consequently, on average, no significant difference is expected in the olfactory deposition.

#### Inspiratory Flow

**Figure 10:**
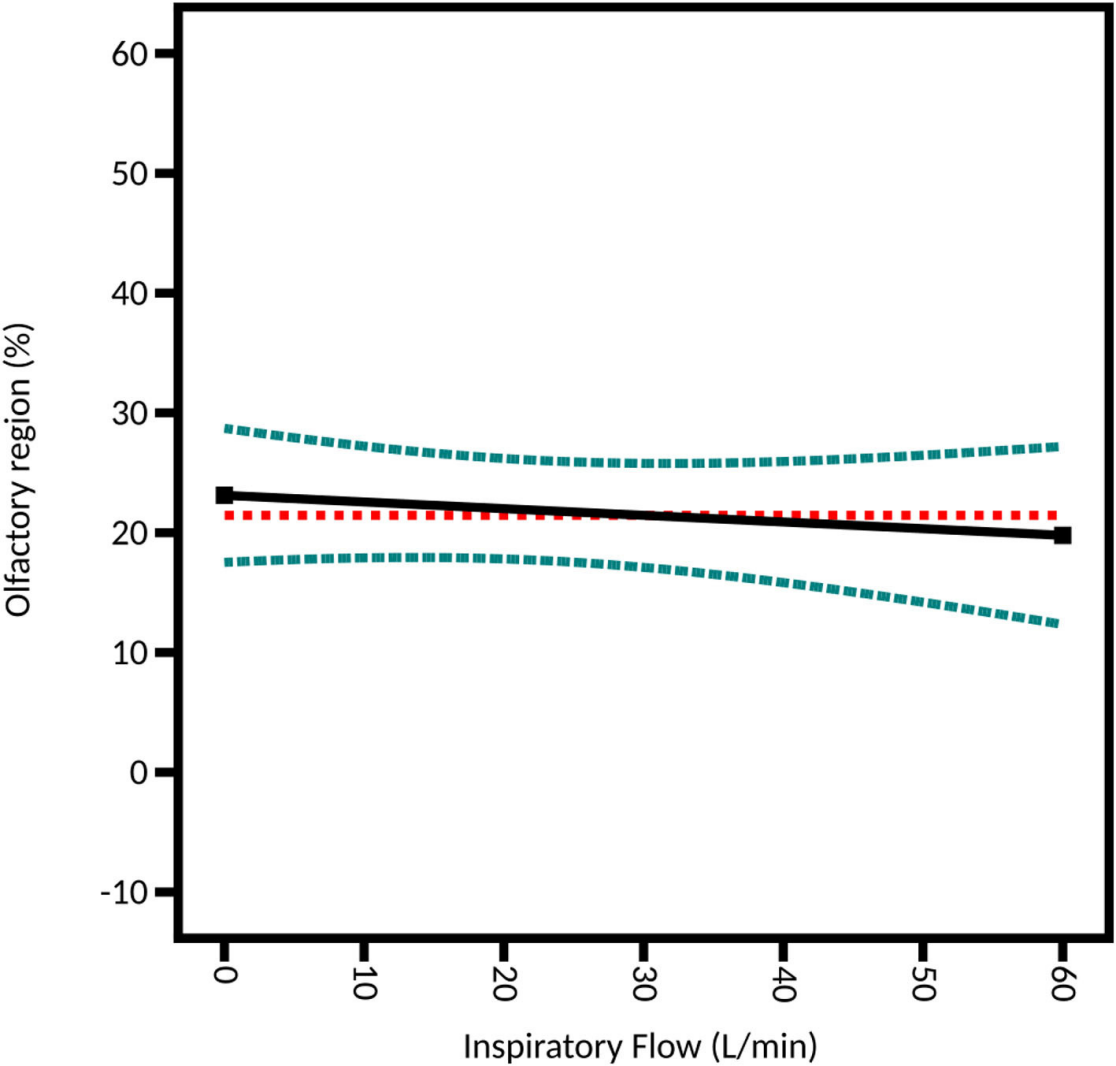
Model graph of the inspiratory flow influence from the DoE focused on the unidirectional device. Results are expressed as the fitted linear model (black solid line) and the 95% confidence interval (blue dashed line).

Studying the influence of a possible concomitant inspiration flow is important since it was shown to affect the trajectory of the spray particles (36). However, it is only possible in the DoE focused on the unidirectional device given that the bidirectional device cannot be used with an inspiration. Three outflows were selected: 0 L/min to simulate a breath-hold, 15 L/min to simulate breathing at rest, and 60 L/min to simulate heavy breathing (12). The difference between the three levels is not significant (*p*-value = 0.6444): 22.09% ± 4.86% of the instilled powder is deposited in the olfactory region on average at 0 L/min, 22.32% ± 6.36% at 15 L/min and 17.49% ± 3.70% at 60 L/min (Figure 10). These results support the recommendation of the manufacturer that says to use the UDS device without inspiration, given that enough thrust is given to the particles by the air pressure generated by the device. These results are also supported by the CFD simulations of Calmet et al. who found that large particles (>10 μm) are trapped in the nasal cavity, no matter the phase of the inspiration (62).

#### Interaction Between the Factors

Over the three DoE, only three interactions are statistically significant: the interaction between the injection nostril and the device, the interaction angle-side-device, and between the septum perforation and the injection nostril for the unidirectional device.

**Figure 11:**
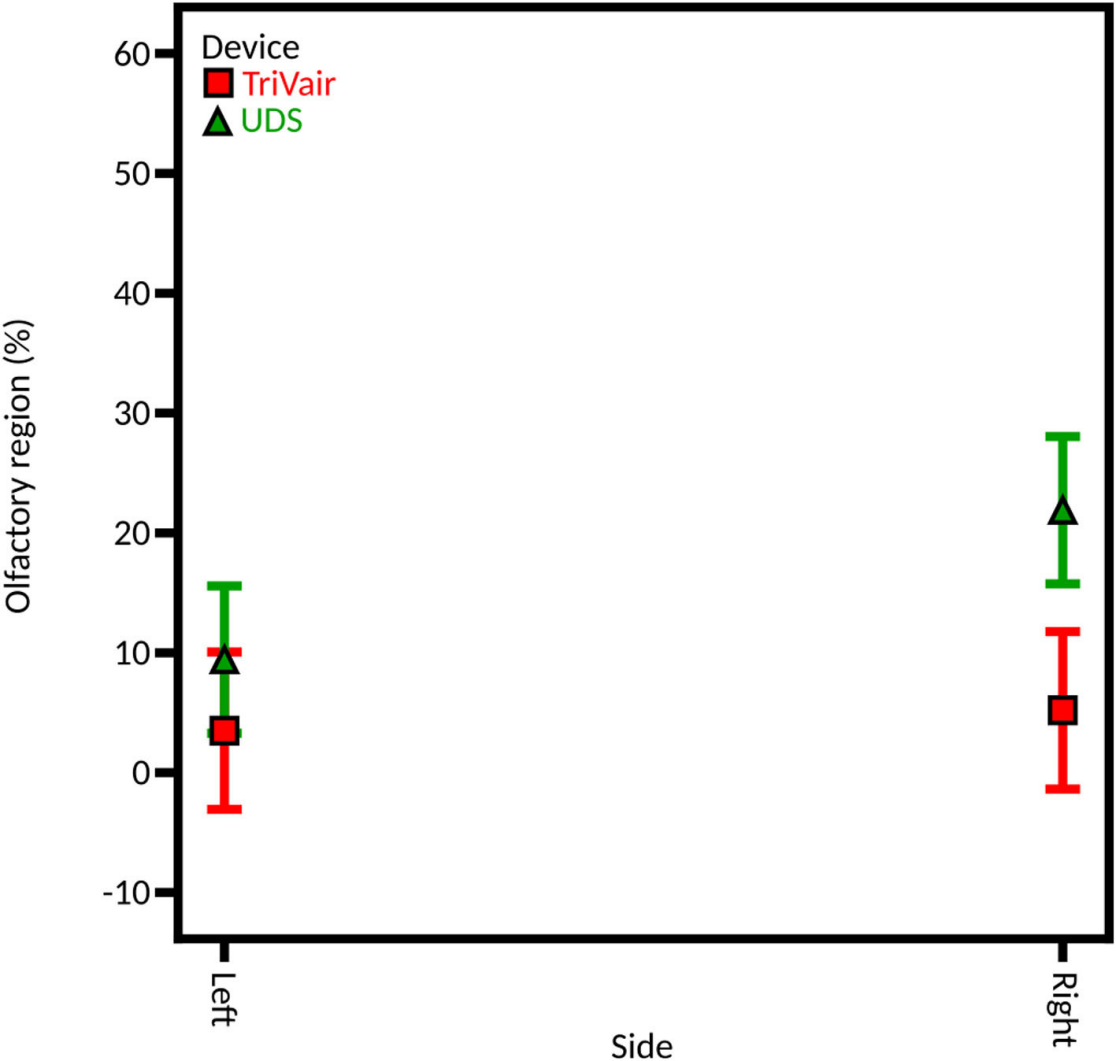
Model graph of the influence of the interaction side-device in olfactory deposition from full DoE. Results are expressed as mean ± RMSE.

First, the interaction side-device influences the deposition beyond the effects of the injection nostril and device (Figure 11). On the left side, the percentage of the instilled powder deposited in the olfactory region is similar for both devices (11.32% ± 2.13% and 6.93% ± 2.38% for unidirectional and bidirectional devices, respectively). However, on the right side, the powder deposition is significantly higher for the unidirectional device than with the bidirectional device (33.35% ± 6.92% and 7.30% ± 2.41%, respectively). It could be due to the right-hand olfactory zone that extends more backward than the left-hand one. Thus, in contrast with the bidirectional device, the unidirectional device was able to reach this area due to its higher ejection velocity but not the bidirectional one. Therefore, the powder deposition increases on the right side with the unidirectional device only.

For the interaction angle-side-device, the powder deposition in the olfactory region obtained with the unidirectional device on the right side with a direct aim is higher than expected when taking each parameter independently. However, there is no visible effect with the bidirectional device.

Finally, a third significant interaction is observed in the DoE focused on the unidirectional device. Without septum perforation, the fraction of the instilled powder deposited on the olfactory region when using the right side is similar to the one obtained using the left side (14.57% ± 5.14% and 17.92% ± 4.59%, respectively). However, with the septum perforation, the deposition is significantly higher on the right side (37.48% ± 5.55% vs. 12.55% ± 4.16%). One reason could be the deflection of the airflow generated by the spray. Indeed, these results suggest that a higher part of the flow crosses the perforation with an instillation on the right side than on the left side and that the flow passing in the perforation brings more particles into the olfactory region.

#### Validation of the Model

**Figure 12:**
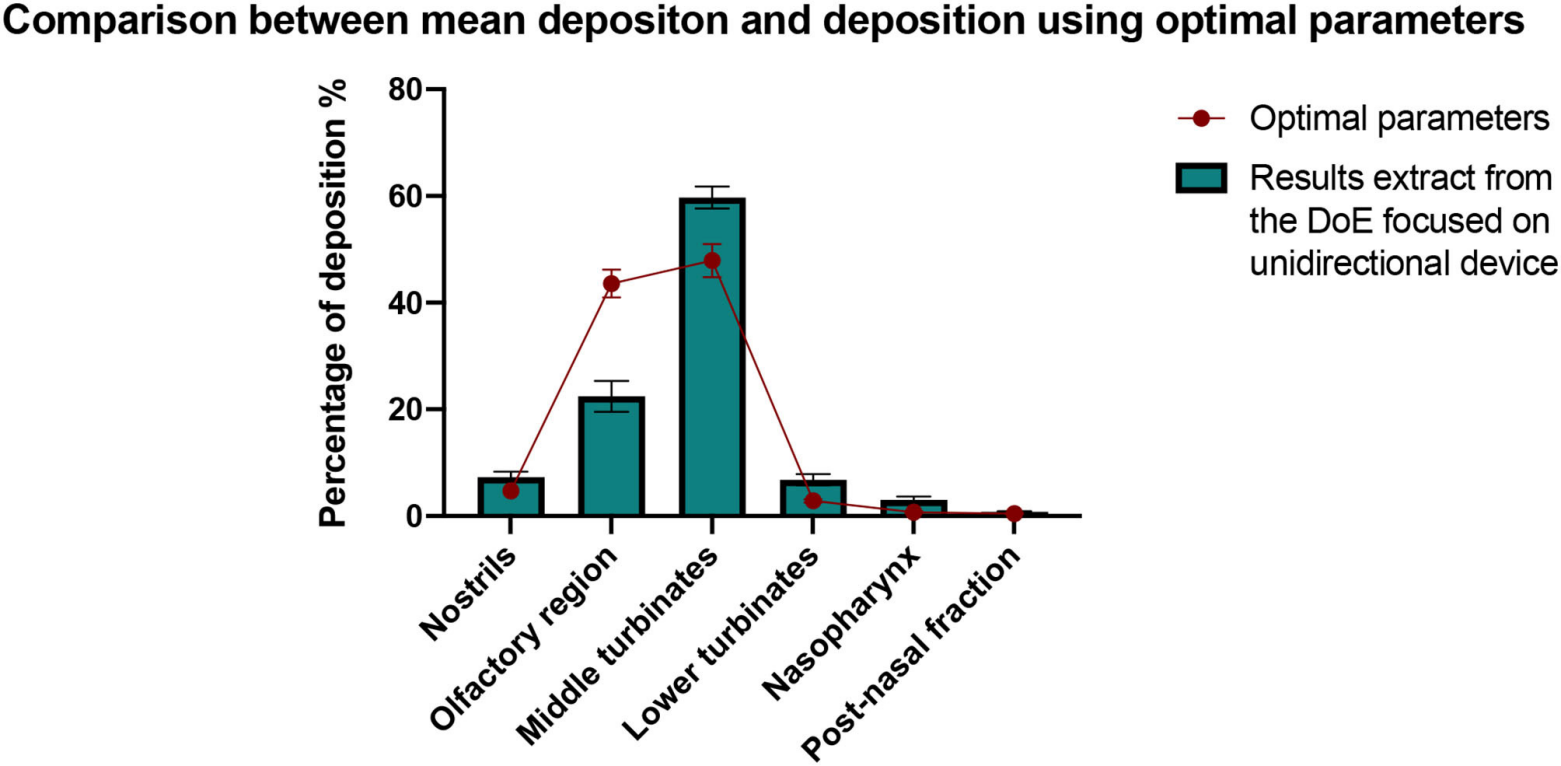
Comparison between the deposition results obtained with the optimal values of the parameters and the mean deposition results from the DoE focused on the unidirectional device. All results are expressed as mean ± SEM.

This study highlights that to optimize the deposition of a powder in the olfactory region with a unidirectional device in this anatomy, the best configuration is to use the device without inspiratory flow, with a direct aim, and in the right nostril. This conclusion is illustrated with the use of predictive points. Indeed, the predictive point which leads to the higher olfactory deposition (direct aim, without inspiratory flow, on the right side with the perforation) was performed in triplicate. As illustrated in Figure 12, when using optimal parameters, the amount of powder deposited in the olfactory region is almost twice larger than the average amount deposited in the different tests of the DoE focused on the unidirectional device (43.6% ± 4.6% and 22.4% ± 15.0%, respectively). The data mean of each region is included in the 95% confidence interval (Table 3). It confirms the robustness of the model.

**Table 3:** Confirmation of the results for the predictive points.

**Analysis**	**Predicted mean**	**Predicted median**	**SD**	***n***	**SE Pred**	**95% PI low**	**Data mean**	**95% PI high**
Olfactory region	51.9988	51.9988	9.193336	3	9.72201	29.5798	43.5667	74.4178

## Conclusion

In conclusion, the use of a 3D-printed nasal cast coated with artificial mucus permitted the determination of the parameters influencing the deposition of a powder in the olfactory region, for unidirectional and bidirectional devices. First, the unidirectional device showed a far better olfactory deposition than the bidirectional one. This effect is probably due to the higher particle ejection velocity generated by the unidirectional device. Then, the deposition in the olfactory region increases by aiming the spray directly at the olfactory region instead of aiming at the center of the nasal valve. The injection nostril and the presence of a perforation in the septum also significantly influences the deposition, suggesting that the efficiency of the treatment can depend on the anatomy of the patient. In contrast, the inspiratory flow did not have a significant effect on powder deposition. By combining these findings, we were able to select the better-performing administration device and the most suitable parameters to use it (i.e., the unidirectional device, instilled in the right nostril without inspiratory flow). This procedure leads to an olfactory deposition efficiency of 44%, which is a great improvement from the mean efficiency of this device (22%). This result highlights the utility of such studies for personalized medicine.

Given the influence of the anatomy observed in this study, a broader study comparing multiple patients could give more precise insights to predict how a spray will deposit in a specific nose. Moreover, the existing publications to confirm some of our hypotheses are scarce, and parameters such as the spray velocity or the dimensions of the cast are rarely reported. Our observations would thus benefit from experimental studies comparing powder devices with diverse characteristics in other anatomies to give a full picture of the phenomenon occurring during spray deposition in the cavity.

## Data Availability Statement

The original contributions presented in the study are included in the article/Supplementary Material, further inquiries can be directed to the corresponding author.

## Ethics Statement

The studies involving human participants were reviewed and approved by the Ethics Committee of Erasme Hospital - ULB. The Ethics Committee waived the requirement of written informed consent for participation.

## Author Contributions

CR and LD contributed to the conception and design of the study, collected the data used in the study, performed the statistical analysis, and wrote the first draft of the manuscript. All authors contributed to manuscript revision, read, and approved the submitted version.

## Conflict of Interest

The authors declare that this study received equipment from AptarGroup and IP Med. The equipment providers were not involved in the study design, collection, analysis, interpretation of data, the writing of this article or the decision to submit it for publication.

## Publisher's Note

All claims expressed in this article are solely those of the authors and do not necessarily represent those of their affiliated organizations, or those of the publisher, the editors and the reviewers. Any product that may be evaluated in this article, or claim that may be made by its manufacturer, is not guaranteed or endorsed by the publisher.
