# Supplementary material for: Instillation of a Dry Powder in Nasal Casts: Parameters Influencing the Olfactory Deposition With Uni- and Bi-Directional Devices
Source: Front Med Technol. 2022 Jun 27;4:924501. doi: 10.3389/fmedt.2022.924501 (PMC9273033; doi:10.3389/fmedt.2022.924501)
Supplement: Supplementary file 1 [file Table_1.DOCX]

***Supplementary data***


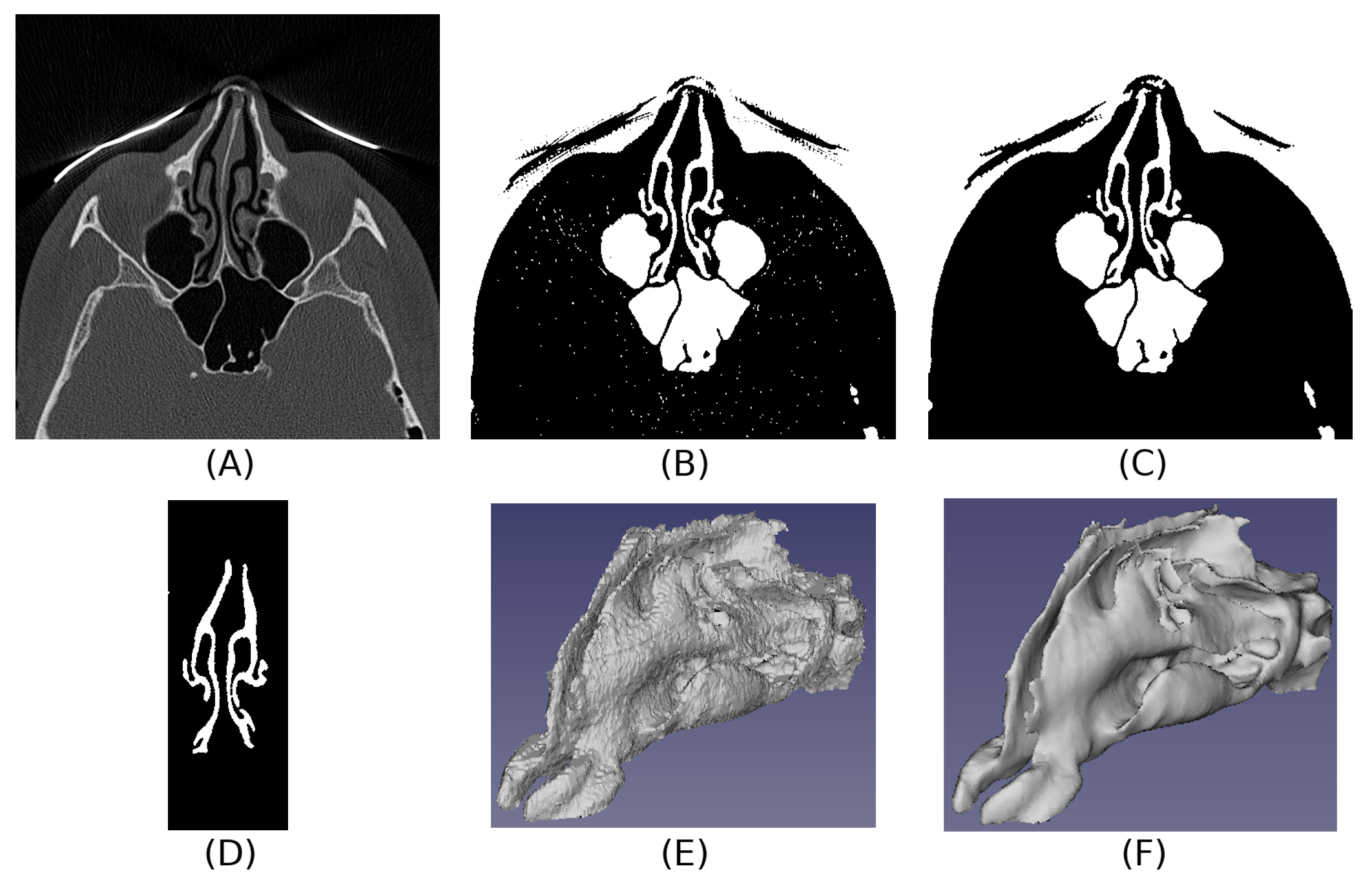


Supplementary Figure 1: Treatment to transform the CT-scan into a 3D geometry of the nasal cavity. (A) original CT-scan; (B) segmentation of the airways; (C) correction of the segmentation; (D) sinuses removal; (E) reconstruction of the geometry; (F) smoothing to obtain the final.


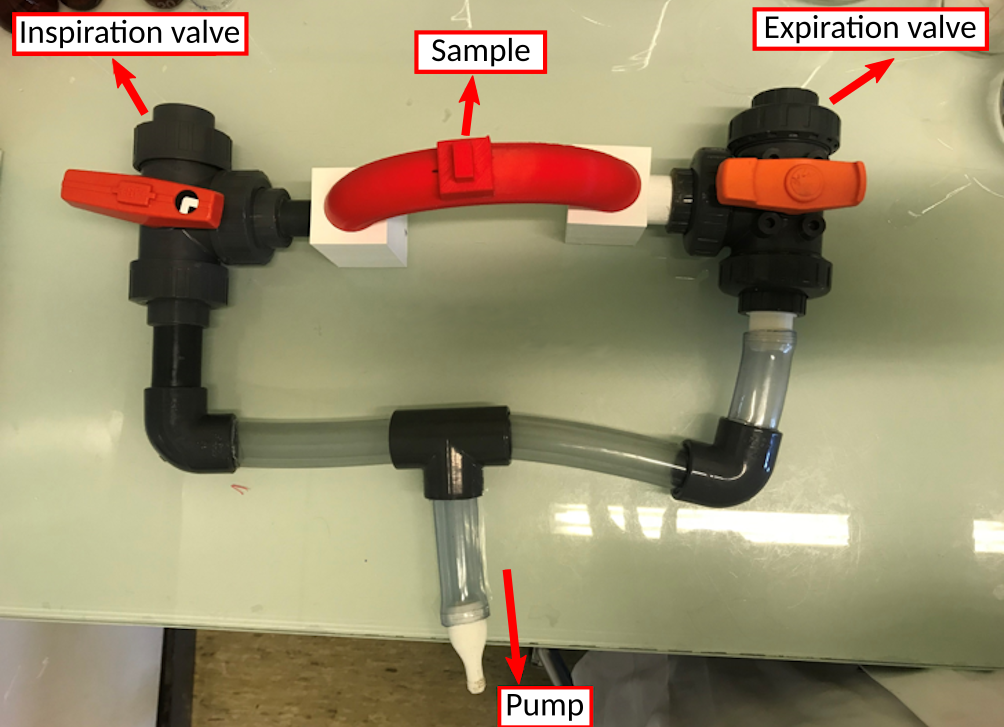
Supplementary Figure 2: Apparatus used to measure the adhesion between the artificial mucus and the powder. A sample is placed on the top of a tube and subjected to alternating flow rate simulating a respiration.


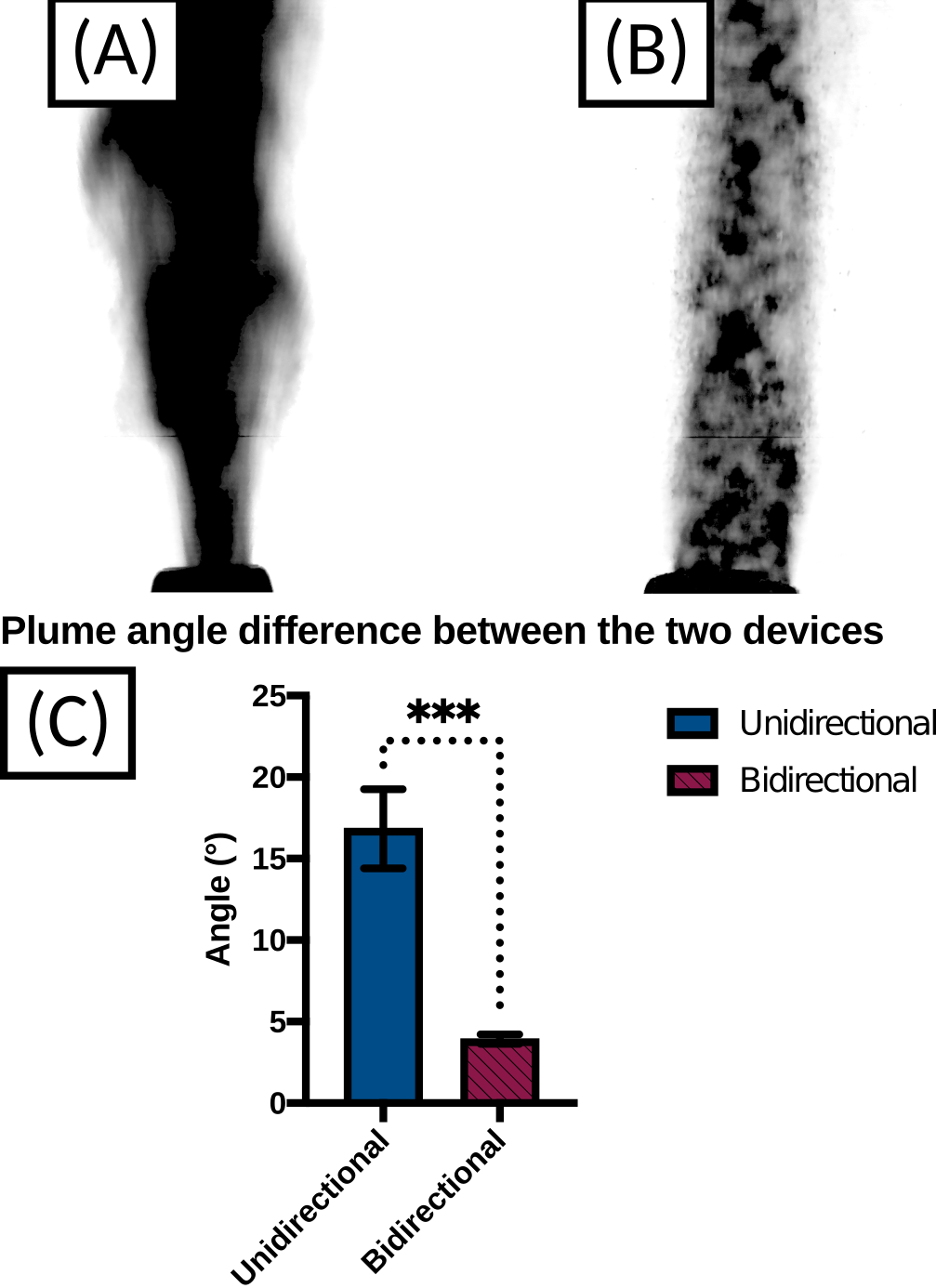


Supplementary Figure 3: (A) Plume angle of the TriVair device, (B) Plume angle of the Aptar device, (C) plume angle difference between the two devices, results were done in triplicate and expressed by mean ± SD (***) p-value < 0.001.


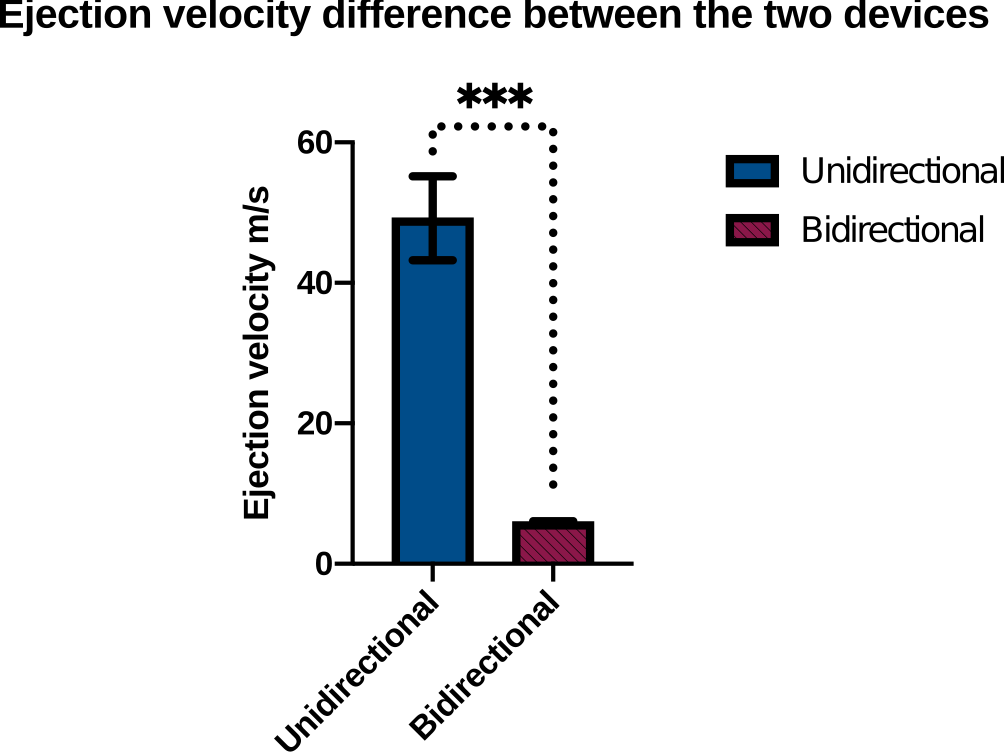


Supplementary Figure 4: Ejection velocity of the Aptar and TriVair devices. Results are expressed in mean ± SD, (***) p-value < 0.001.

Supplementary Table 1: Categoric factors of the full DoE.

| **Categoric factors** | **Level [1]** | **Level [2]** |
| --- | --- | --- |
| A: Angle | Center | Direct |
| B: Perforation | Without | With |
| C: Side | Left | Right |
| D: Device | Bidirectional | Unidirectional |

Supplementary Table 2: Categoric factors of the DoE focused on unidirectional device.

| **Categoric factors** | **Level [1]** | **Level [2]** | **Level [3]** |
| --- | --- | --- | --- |
| A: Inspiratory flow (L/min) | 0 | 15 | 60 |
| B: Angle | Center | Direct | - |
| C: Perforation | Without | With | - |
| D: Side | Left | Right | - |

Supplementary Table 3: Categoric factors of the DoE focused on bidirectional device.

| **Categoric factors** | **Level [1]** | **Level [2]** |
| --- | --- | --- |
| Angle | Center | Direct |
| Perforation | Without | With |
| Side | Left | Right |

| **Source** | **Sum of Squares** | **df** | **Mean Square** | **F-value** | **p-value** |  |
| --- | --- | --- | --- | --- | --- | --- |
| **Model** | 2975.57 | 8 | 371.95 | 9.56 | 0.0009 | significant |
| **A-Angle** | 414.72 | 1 | 414.72 | 10.66 | 0.0085 |  |
| **B-Perforation** | 112.50 | 1 | 112.50 | 2.89 | 0.1199 |  |
| **C-Side** | 598.27 | 1 | 598.27 | 15.37 | 0.0029 |  |
| **D-Device** | 1112.74 | 1 | 1112.74 | 28.60 | 0.0003 |  |
| **AC** | 87.01 | 1 | 87.01 | 2.24 | 0.1657 |  |
| **AD** | 88.68 | 1 | 88.68 | 2.28 | 0.1621 |  |
| **CD** | 593.92 | 1 | 593.92 | 15.26 | 0.0029 |  |
| **ACD** | 165.65 | 1 | 165.65 | 4.26 | 0.0660 |  |
| **Residual** | 389.12 | 10 | 38.91 |  |  |  |
| **Lack of Fit** | 271.68 | 7 | 38.81 | 0.9914 | 0.5562 | not significant |
| **Pure Error** | 117.44 | 3 | 39.15 |  |  |  |
| **Cor Total** | 3364.70 | 18 |  |  |  |  |

Supplementary Table 4: ANOVA table extracted from the full DoE.

Supplementary Table 5: ANOVA table extracted from the DoE focused on unidirectional device.

| **Source** | **Sum of Squares** | **df** | **Mean Square** | **F-value** | **p-value** |  |
| --- | --- | --- | --- | --- | --- | --- |
| **Model** | 3680.86 | 5 | 736.17 | 7.16 | 0.0005 | significant |
| **A-Inspiratory flow** | 51.05 | 1 | 51.05 | 0.4963 | 0.4889 |  |
| **B-Angle** | 1388.12 | 1 | 1388.12 | 13.50 | 0.0014 |  |
| **C-Perforation** | 255.56 | 1 | 255.56 | 2.48 | 0.1299 |  |
| **D-Side** | 1407.74 | 1 | 1407.74 | 13.69 | 0.0013 |  |
| **CD** | 593.70 | 1 | 593.70 | 5.77 | 0.0256 |  |
| **Residual** | 2160.00 | 21 | 102.86 |  |  |  |
| **Lack of Fit** | 1543.52 | 16 | 96.47 | 0.7824 | 0.6789 | not significant |
| **Pure Error** | 616.47 | 5 | 123.29 |  |  |  |
| **Cor Total** | 5840.86 | 26 |  |  |  |  |

Supplementary Table 6: ANOVA table extracted from the DoE focused on bidirectional device.

| **Source** | **Sum of Squares** | **df** | **Mean Square** | **F-value** | **p-value** |  |
| --- | --- | --- | --- | --- | --- | --- |
| **Model** | 47.11 | 3 | 15.70 | 0.5710 | 0.6581 | not significant |
| **A-Angle** | 47.01 | 1 | 47.01 | 1.71 | 0.2480 |  |
| **B-Perforation** | 0.8074 | 1 | 0.8074 | 0.0294 | 0.8707 |  |
| **C-Side** | 0.5638 | 1 | 0.5638 | 0.0205 | 0.8917 |  |
| **Residual** | 137.51 | 5 | 27.50 |  |  |  |
| **Lack of Fit** | 113.01 | 4 | 28.25 | 1.15 | 0.5955 | not significant |
| **Pure Error** | 24.50 | 1 | 24.50 |  |  |  |
| **Cor Total** | 184.62 | 8 |  |  |  |  |


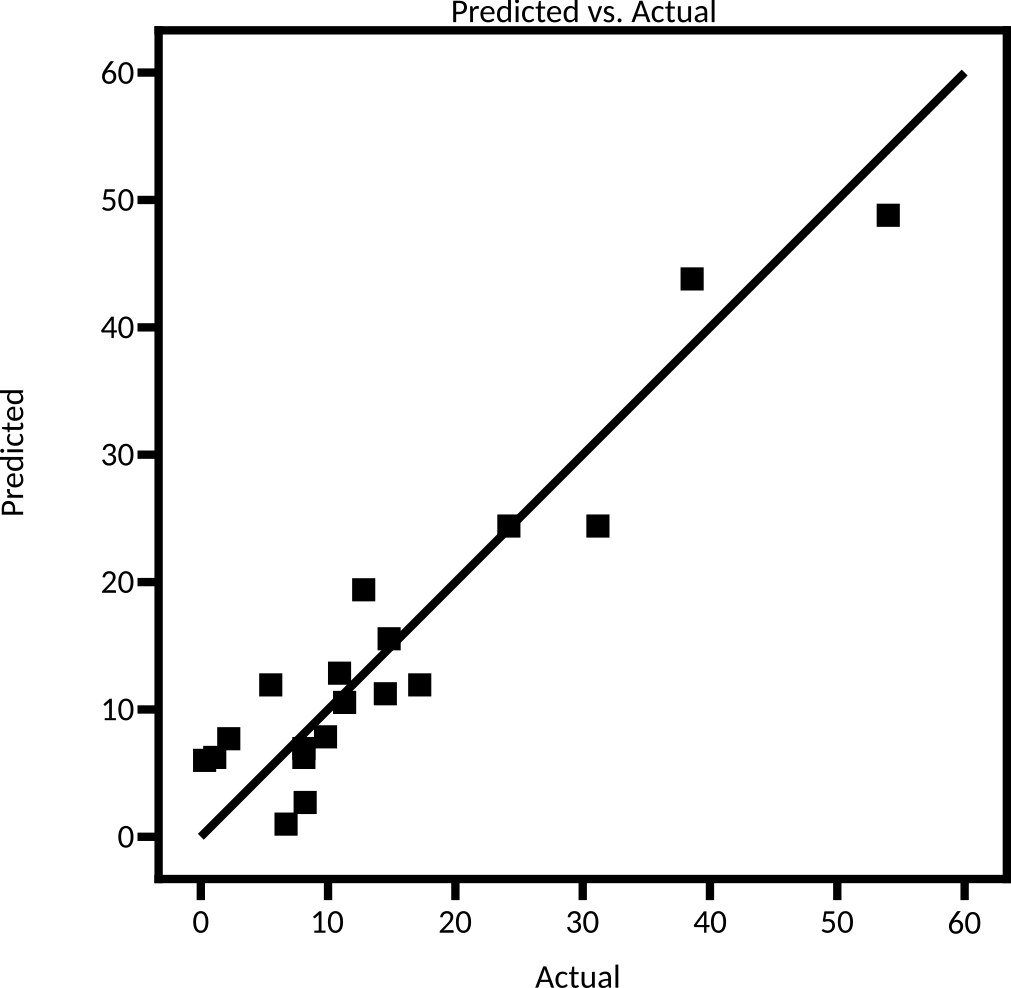


Supplementary Figure 5: Experimental values of olfactory deposition (colored squares) and model (black line) extracted from the full DoE.

Supplementary Table 7 : Coefficients of the model fitted on the full DoE.

| ***Factor*** | ***Coefficient Estimate*** | ***df*** | ***Standard Error*** | ***95% CI Low*** | ***95% CI High*** | ***VIF*** |
| --- | --- | --- | --- | --- | --- | --- |
| Intercept | 14.81 | 1 | 1.46 | 11.56 | 18.06 |  |
| A-Angle | 4.80 | 1 | 1.47 | 1.52 | 8.08 | 1.05 |
| B-Perforation | 2.50 | 1 | 1.47 | -0.7760 | 5.78 | 1.05 |
| C-Side | 5.72 | 1 | 1.46 | 2.47 | 8.98 | 1.04 |
| D-Device | 7.86 | 1 | 1.47 | 4.59 | 11.14 | 1.05 |
| AC | 2.18 | 1 | 1.46 | -1.07 | 5.44 | 1.04 |
| AD | 2.20 | 1 | 1.46 | -1.05 | 5.46 | 1.01 |
| CD | 5.70 | 1 | 1.46 | 2.45 | 8.96 | 1.04 |
| ACD | 3.01 | 1 | 1.46 | -0.2407 | 6.27 | 1.04 |
|  |  |  |  |  |  |  |


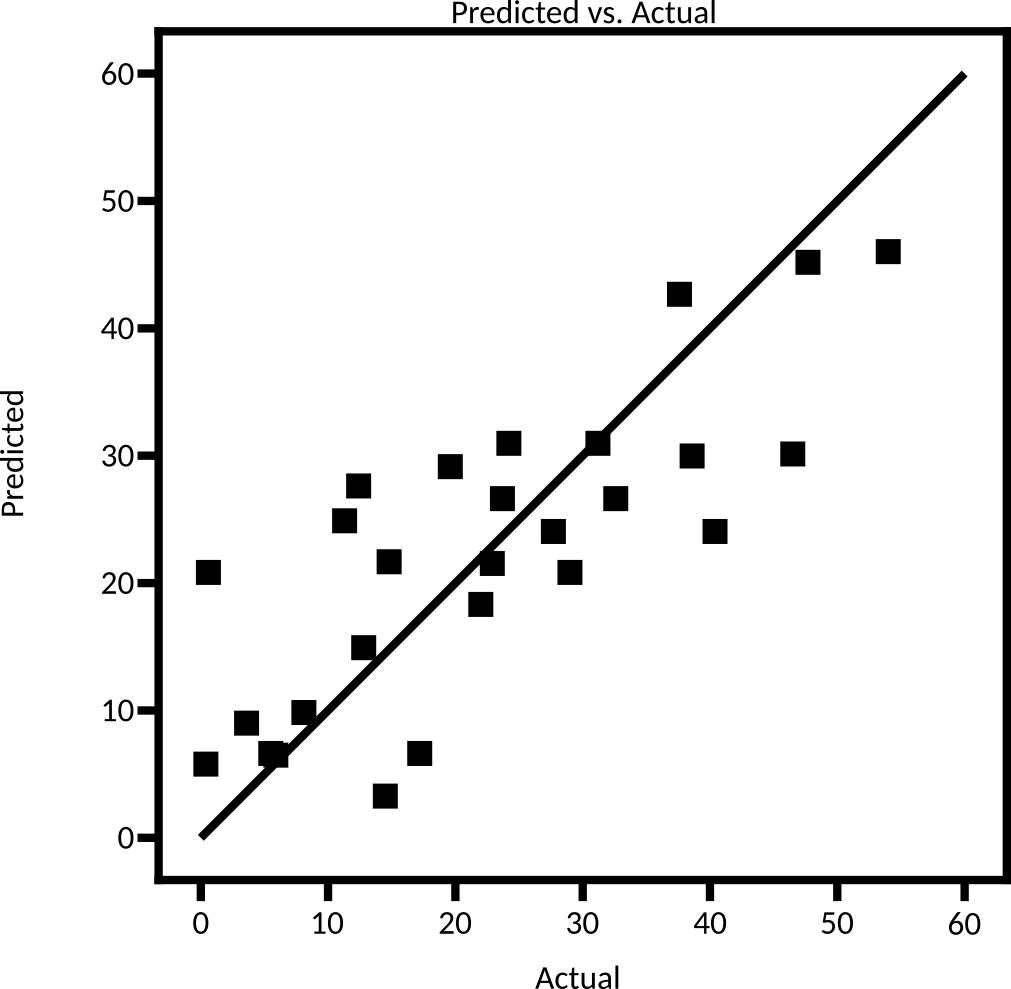


Supplementary Figure 6: Experimental values of olfactory deposition and model extracted from the DoE focused on unidirectional device.

Supplementary Table 8: Coefficients of the model fitted on the DoE focused on unidirectional device.

| ***Factor*** | ***Coefficient Estimate*** | ***df*** | ***Standard Error*** | ***95% CI Low*** | ***95% CI High*** | ***VIF*** |
| --- | --- | --- | --- | --- | --- | --- |
| Intercept | 21.44 | 1 | 2.09 | 17.10 | 25.78 |  |
| A-Inspiratory flow | -1.67 | 1 | 2.38 | -6.61 | 3.27 | 1.03 |
| B-Angle | 7.53 | 1 | 2.05 | 3.27 | 11.79 | 1.09 |
| C-Perforation | 3.21 | 1 | 2.03 | -1.02 | 7.44 | 1.07 |
| D-Side | 7.36 | 1 | 1.99 | 3.22 | 11.49 | 1.03 |
| CD | 4.82 | 1 | 2.00 | 0.6473 | 8.99 | 1.05 |


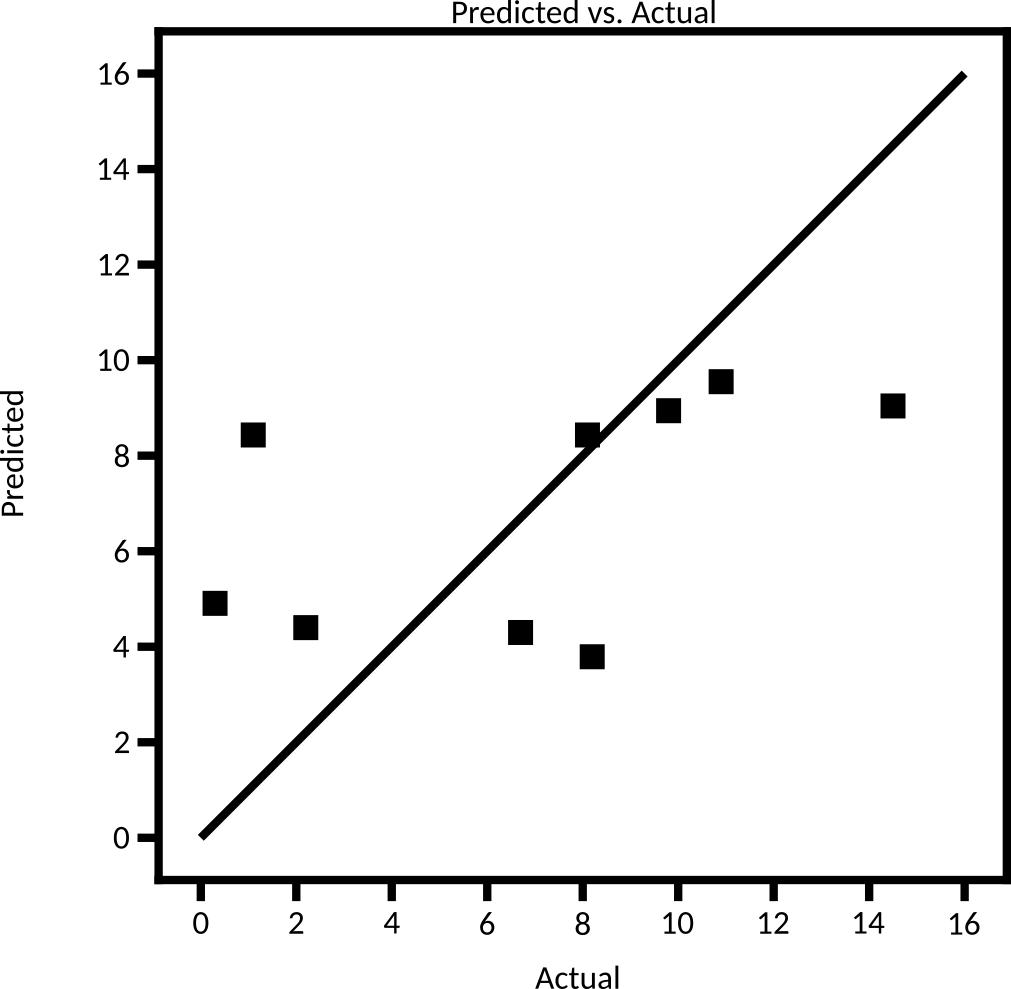


Supplementary Figure 7: Experimental values of olfactory deposition (colored squares) and model (black line) extracted from the DoE focused on bidirectional device.

Supplementary Table 9: Coefficients of the model fitted on the DoE focused on bidirectional device.

| ***Factor*** | ***Coefficient Estimate*** | ***df*** | ***Standard Error*** | ***95% CI Low*** | ***95% CI High*** | ***VIF*** |
| --- | --- | --- | --- | --- | --- | --- |
| Intercept | 6.67 | 1 | 1.78 | 2.11 | 11.23 |  |
| A-Angle | 2.32 | 1 | 1.78 | -2.24 | 6.88 | 1.02 |
| B-Perforation | 0.3042 | 1 | 1.78 | -4.26 | 4.87 | 1.02 |
| C-Side | -0.2542 | 1 | 1.78 | -4.82 | 4.31 | 1.02 |


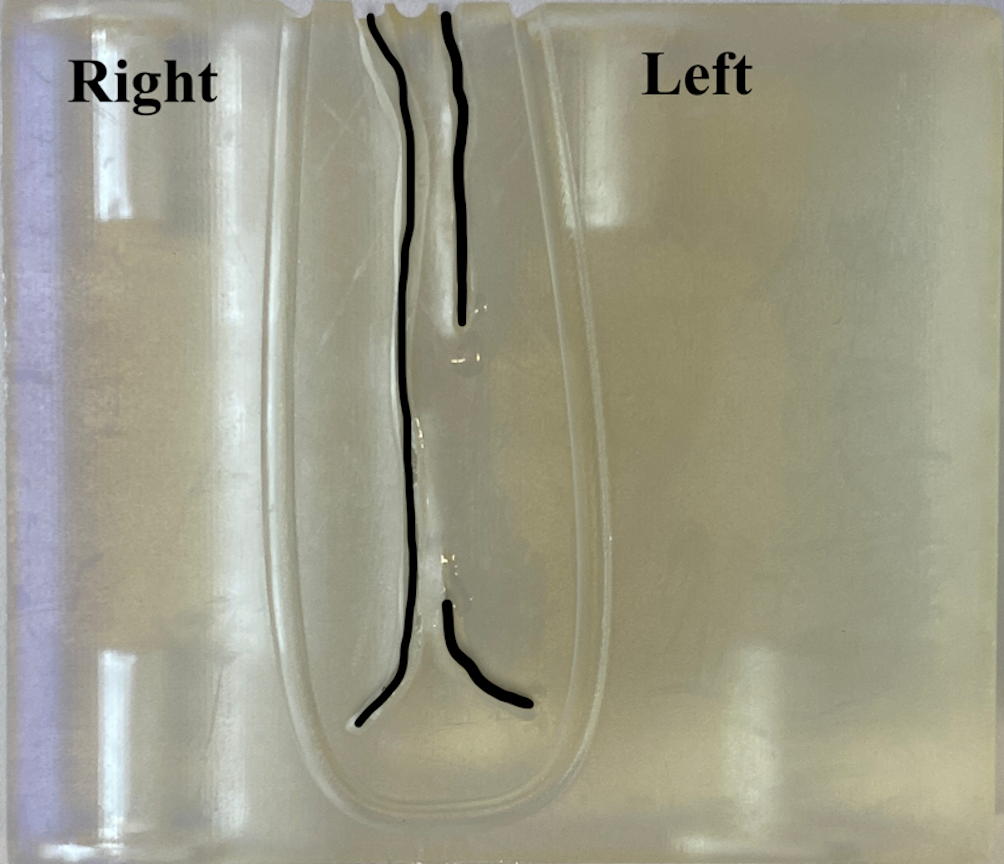


Supplementary Figure 8: Highlight of the surface of the olfactory region in right and left side.


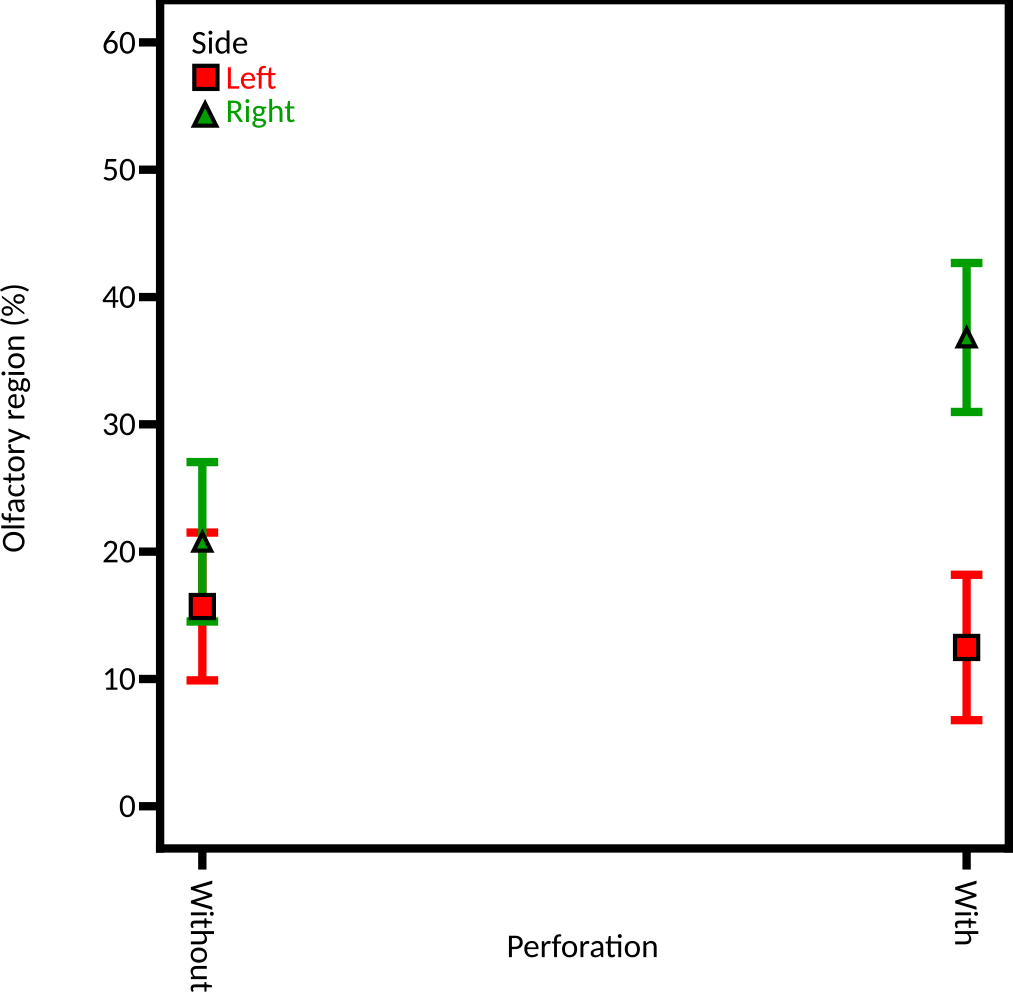


Supplementary Figure 9: Model graph of the influence of the interaction side-device in olfactory deposition from full DoE. Results are expressed by mean ± RMSE.


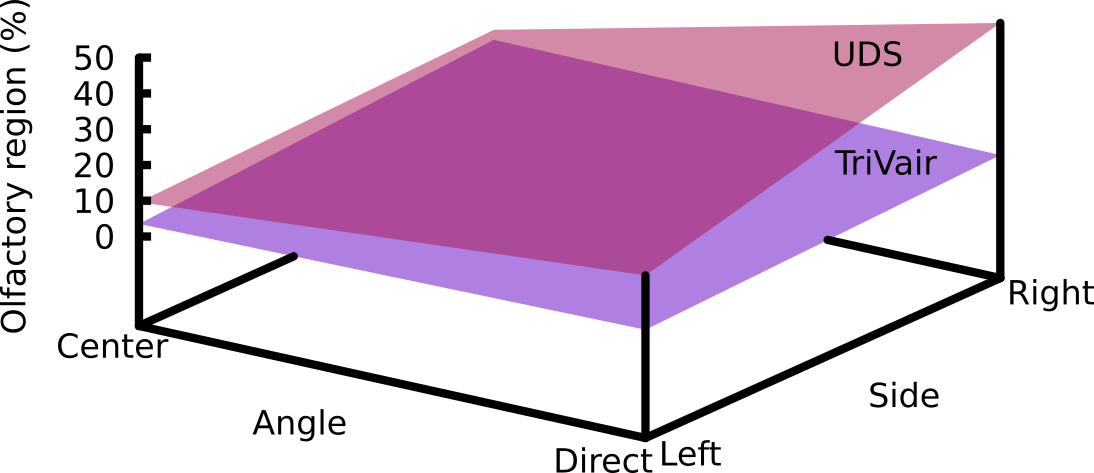


Supplementary Figure 10: Model graph of the influence of the interaction angle-side-device in olfactory deposition from full DoE.
